# T-Cell–Derived miRNA-214 Mediates Perivascular Fibrosis in Hypertension

**DOI:** 10.1161/CIRCRESAHA.119.315428

**Published:** 2020-02-17

**Authors:** Ryszard Nosalski, Mateusz Siedlinski, Laura Denby, Eilidh McGinnigle, Michal Nowak, Aurelie Nguyen Dinh Cat, Laura Medina-Ruiz, Marco Cantini, Dominik Skiba, Grzegorz Wilk, Grzegorz Osmenda, Julie Rodor, Manuel Salmeron-Sanchez, Gerard Graham, Pasquale Maffia, Delyth Graham, Andrew H. Baker, Tomasz J. Guzik

**Affiliations:** 1 From the Institute of Cardiovascular and Medical Sciences, University of Glasgow, United Kingdom (R.N., E.M., A.N.D.C., D.S., P.M., D.G., T.J.G.); 2 Department of Medicine, Jagiellonian University Medical College, Krakow, Poland (R.N., M.S., M.N., D.S., G.W., G.O., T.J.G.); 3 Centre for Cardiovascular Science, Queen’s Medical Research Institute, University of Edinburgh, United Kingdom (L.D., J.R., A.H.B.); 4 Institute of Infection, Immunity and Inflammation, University of Glasgow, United Kingdom (L.M.-R., G.G., P.M.); 5 Centre for the Cellular Microenvironment, School of Engineering, University of Glasgow, United Kingdom (M.C., M.S.-S.); 6 Department of Pharmacy, University of Naples Federico II, Italy (P.M.).

**Keywords:** blood pressure, collagen, fibrosis, hypertension, inflammation

## Abstract

**Rationale:**

Despite increasing understanding of the prognostic importance of vascular stiffening linked to perivascular fibrosis in hypertension, the molecular and cellular regulation of this process is poorly understood.

**Objectives:**

To study the functional role of microRNA-214 (miR-214) in the induction of perivascular fibrosis and endothelial dysfunction driving vascular stiffening.

**Methods and Results:**

Out of 381 miRs screened in the perivascular tissues in response to Ang II (angiotensin II)-mediated hypertension, miR-214 showed the highest induction (8-fold, *P*=0.0001). MiR-214 induction was pronounced in perivascular and circulating T cells, but not in perivascular adipose tissue adipocytes. Global deletion of miR-214^−^^/−^ prevented Ang II-induced periaortic fibrosis, *Col1a1*, *Col3a1, Col5a1*, and *Tgfb1* expression, hydroxyproline accumulation, and vascular stiffening, without difference in blood pressure. Mechanistic studies revealed that miR-214^−/−^ mice were protected against endothelial dysfunction, oxidative stress, and increased Nox2, all of which were induced by Ang II in WT mice. Ang II-induced recruitment of T cells into perivascular adipose tissue was abolished in miR-214^−/−^ mice. Adoptive transfer of miR-214^−/−^ T cells into RAG1^−/−^ mice resulted in reduced perivascular fibrosis compared with the effect of WT T cells. Ang II induced hypertension caused significant change in the expression of 1380 T cell genes in WT, but only 51 in miR-214^−/−^. T cell activation, proliferation and chemotaxis pathways were differentially affected. MiR-214^−/−^ prevented Ang II-induction of profibrotic T cell cytokines (*IL-17*, *TNF-α, IL-9*, and *IFN-γ*) and chemokine receptors (CCR1, CCR2, CCR4, CCR5, CCR6, and CXCR3). This manifested in reduced in vitro and in vivo T cell chemotaxis resulting in attenuation of profibrotic perivascular inflammation. Translationally, we show that miR-214 is increased in plasma of patients with hypertension and is directly correlated to pulse wave velocity as a measure of vascular stiffness.

**Conclusions:**

T-cell–derived miR-214 controls pathological perivascular fibrosis in hypertension mediated by T cell recruitment and local profibrotic cytokine release.

## Abstract 2

Supplemental Digital Content is available in the text.

**Editorial, see p 1004**

**Meet the First Author, see p 944**

Hypertension continues to be the number one cause of death and disability worldwide.^1^ While blood pressure reduction improves disease outcomes, cardiovascular risk remains highly elevated indicating that underlying pathological processes are essential in mediating adverse clinical outcomes. Accelerated vascular aging a hallmark of hypertension underlies all of the target organ consequences of the disease.^2^ It encompasses a number of pathophysiologic processes including increased vascular oxidative stress, increased perivascular inflammation, endothelial dysfunction, all leading collectively to augmentation of vascular stiffening mediated primarily by perivascular fibrosis.^3–6^ Vascular stiffening, measured by pulse wave velocity (PWV) is uniformly accepted as an important predictor of cardiovascular morbidity and mortality.^1,7^ Therefore, understanding the molecular mechanisms of vascular stiffening is essential for development of novel therapies which can to reduce cardiovascular risk in hypertension. Inflammation has been one of the important common denominators of cardiovascular risk^8^ and arterial stiffness^9^ and immune cell infiltration is pivotal for perivascular fibrosis.^6,10–12^

While the opposing effects of miR-214 have been recently defined in cardiac^13,14^ and renal^15,16^ fibrosis, the expression and function of miR-214 in vascular fibrosis and stiffening remains unknown. Perivascular fibrosis is largely dependent on perivascular inflammation.^6^ Notably, miR-214 is expressed by immune cells and affects their functions as indicated by studies of tumor immunity^17^ including possible regulation of profibrotic cytokines, such as TGF-β (transforming growth factor-β) or IL (interleukin)-17.^18^ This is important as IL-17-mediated vascular stiffening can occur through increasing the deposition of collagen.^10^

We sought to examine mechanisms responsible for aortic stiffening in hypertension focusing on the role of microRNAs (miRs) in the regulation of vascular fibrosis. Recent studies have shown that perivascular inflammation regulates vascular dysfunction and remodeling in hypertension.^10,19^ Here, we have identified that miR-214 is increased in perivascular adipose tissue (PVAT) in hypertension, and that this increase is observed primarily in T cells infiltrating the vessel. MiR-214 expression in T cells enables their activation and recruitment into perivascular fat in the context of hypertension. It also regulates T cell profibrotic properties in response to prohypertensive stimuli. Translational relevance of these findings has been confirmed by the fact that plasma miR-214 is increased in hypertension and is directly correlated to PWV and endothelial function.

## Methods

The data that support the findings of this study are available from the first and corresponding authors upon reasonable request.

The hypertension and perivascular inflammation studies have been conducted as described previously.^19,20^ Hypertension was induced in 12-week-old male mice by infusion of Ang II (angiotensin II; 490 ng/kg per day) for 14 days via osmotic minipumps (Model 2002, Alzet Corporation). Male mice were used as this is best validated model for hypertension, particularly in the context of T cell immune responses. C57BL/6 and Rag1^−/−^ (B6.129S7-Rag1tm1Mom/J) were obtained from Jackson Laboratories. The miR-214^−/−^ mice were kindly gifted by Dr Eric Olson previously described which for adoptive transfer experiments were backcrossed 7× to C57BL/6 N background. All animal experiments were performed in accordance with the United Kingdom Animals Scientific Procedures Act 1986 and ARRIVE (Animal Research: Reporting of In Vivo Experiments) Guidelines and approved by the Home Office (No. 7009021) and ethics committee of Jagiellonian University (157/2016). All experiments conform the guidelines from Directive 2010/63/EU of the European Parliament on the protection of animals used for scientific purposes. Randomization and allocation concealment were performed. Kolmogorov-Smirnov test, Shapiro-Wilk, q-q-plots, and histograms were used to test normality of data distribution before parametric or nonparametric tests were applied. Correlation between cells was assessed by Spearman rank correlation analysis. Statistical analysis was performed using *t*-test (2-group studies), 1- or 2-way ANOVA (>2-group studies) with a Tukey multiple comparison test, Kruskal-Wallis test, or Mann-Whitney *U* test. Data are reported as the mean±SEM. *P* values <0.05 were considered significant. Reported *P* values include multiple comparison correction (Bonferroni or FDR). Number of samples is given in individual Figure legends and represent biological replicates (mice used for experiments). Data based on tail cuff experiments indicate that to detect SBP change from 123 to 143 mm Hg, 8 mice/group are needed to detect significant (*P*<0.05) difference at a power of 80%. Differential gene expression analysis from the RNA-Seq experiment was performed using DESeq2 package in R (ver. 3.5.1). Raw gene counts of the RNA-Seq analysis are available as supplemental material. RNA-Seq datasets are deposited in the Gene Expression Omnibus (GEO)-accession number GSE143809 database.

Translational study was performed on 51 hypertensive and 49 normotensive subjects (Clinical Characteristics—Online Table I) and included analysis of miR-214 in plasma, PWV, and flow-mediated dilatation studies as described previuosly.^19,21^ Study was performed according to the Declaration of Helsinki and was approved by the Ethics Committee of Jagiellonian University. All participants gave written informed consent.

Analysis in patient populations were performed in Statistica 13 TIBCO using *t* test for independent samples. Correlation between PWV and plasma miR-214 was assessed by Spearman rank correlation test and then by multiple regression correcting for SBP and age.

A detailed Materials and Methods section is provided in the Data Supplement.

## Results

### miR-214 Is Increased in the Vasculature in Ang II-Induced Hypertension

**Figure 1.**
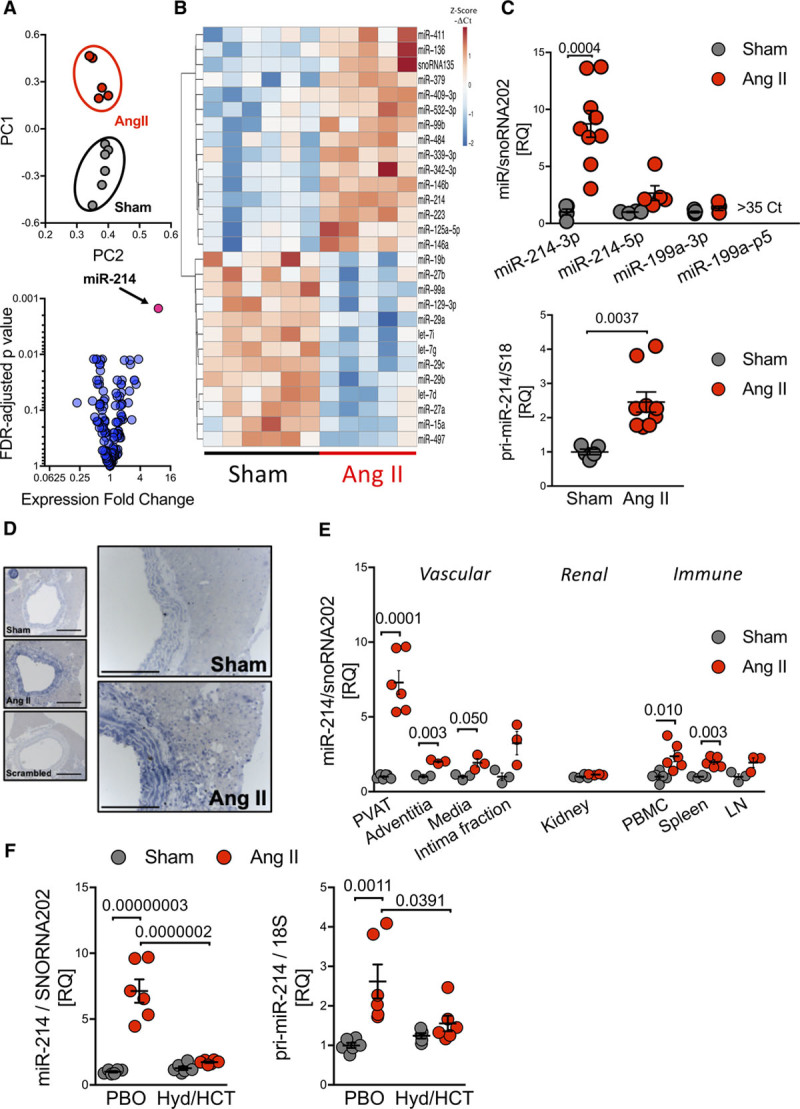
**MiR-214 is increased in the vasculature and immune cells in Ang II (angiotensin II)-induced hypertension.** **A**, Principal component analysis of miRs in perivascular adipose tissue (PVAT; top) and volcano plot (bottom) in sham and Ang II-treated mice. **B**, Heatmap presenting *z*-scores calculated from individual miR −ΔCt values of all miRs significantly altered (*t*-test FDR adj *P*<0.05; n=5–6). **C**, Levels of miR-214/199 cluster (top) and pri-miR-214 (bottom) in PVAT (n=4–9). **D**, In situ hybridization of miR-214 in the vascular wall. Scale bar=300 and 150 *μ*m. **E**, Levels of miR-214 in isolated PVAT, vessel wall layers (n=3 replicates of 3 pulled mice—9 mice/group), kidney, and selected immune organs (n=3–6). **F**, Levels of miR-214 and pri-miR-214 in PVAT of mice treated with placebo (PBO) or hydralazine/hydrocholrothiazide (Hyd/HCT; n=6/group). Data presented as mean±SEM; *t*-test with FDR correction (**C**, *P* values adjusted for 4 comparisons; miR clusters; **E**, *P* values adjusted for 8 comparisons; various organs) or by 2-way ANOVA (for miR-214 level; *P*^AngII×Drug^=3.7×10^−6^, *P*^AngII^=4.3×10^−7^, *P*^Drug^=1.4×10^−5^; for pri-miR-214 level; *P*^AngII×Drug^=0.018, *P*^AngII^=1.2×10^−3^, *P*^Drug^=0.12) with Tukey multiple comparisons test (**F**; *P* values adjusted for 6 comparisons).

To identify miR potentially important in the regulation of perivascular inflammation and dysfunction in hypertension, we performed a TaqMan array analysis in perivascular tissue of sham- and Ang II-infused mice. Principal component analysis comprising miR transcripts detected a notable separation of sample sets (Figure 1A, top). Of 381 assayed miR, 134 showed expression below 35th Ct value. Twenty eight showed differential expression (Figure 1A and 1B). Fifteen were significantly upregulated and 13 were downregulated (Figure 1B). MiR-214 was the only over-expressed miR significantly altered after Bonferroni correction showing an 8-fold increase (Figure 1A and 1B), which was not observed in other members of miR-199/214 cluster (Figure 1C, top). Similar, although less pronounced an increase was observed in the pri-miR-214 transcript (Figure 1C, bottom). In situ hybridization (Figure 1D) and RT-PCR (Figure 1E) was used to visualize and quantify the induction of miR-214 in different areas of the vessel. MiR-214 was increased nearly 8-fold in PVAT and only 2-fold in other vessel layers. Surprisingly, no increase was observed in primary adipocytes isolated from PVAT of Ang II-treated versus sham mice (Online Figure I). Subsequent interrogation of the effects of Ang II on miR-214 expression in kidneys, peripheral blood mononuclear cells, spleen, and lymph nodes demonstrated its pronounced increase in the spleen and peripheral blood mononuclear cells (Figure 1E).

To investigate if the miR-214 increase in the PVAT was evoked by the direct actions of Ang II or indirectly by blood pressure elevation we used the model of pretreatment of mice with oral hydralazine (Hyd) and hydrochlorothiazide (HCT),^11^ which reduced blood pressure in spite of Ang II infusion from 165±5 mm Hg (placebo; Ang II) to 133±6 mm Hg (Hyd/HCT; Ang II; Online Figure II). The increase in PVAT miR-214 (Figure 1F, left) and pri-miR-214 (Figure 1F, right) was abolished in Hyd/HCT mice in spite of Ang II infusion.

### miR-214^−/−^ Protects From Hypertension-Induced Vascular Stiffness and Perivascular Fibrosis

**Figure 2.**
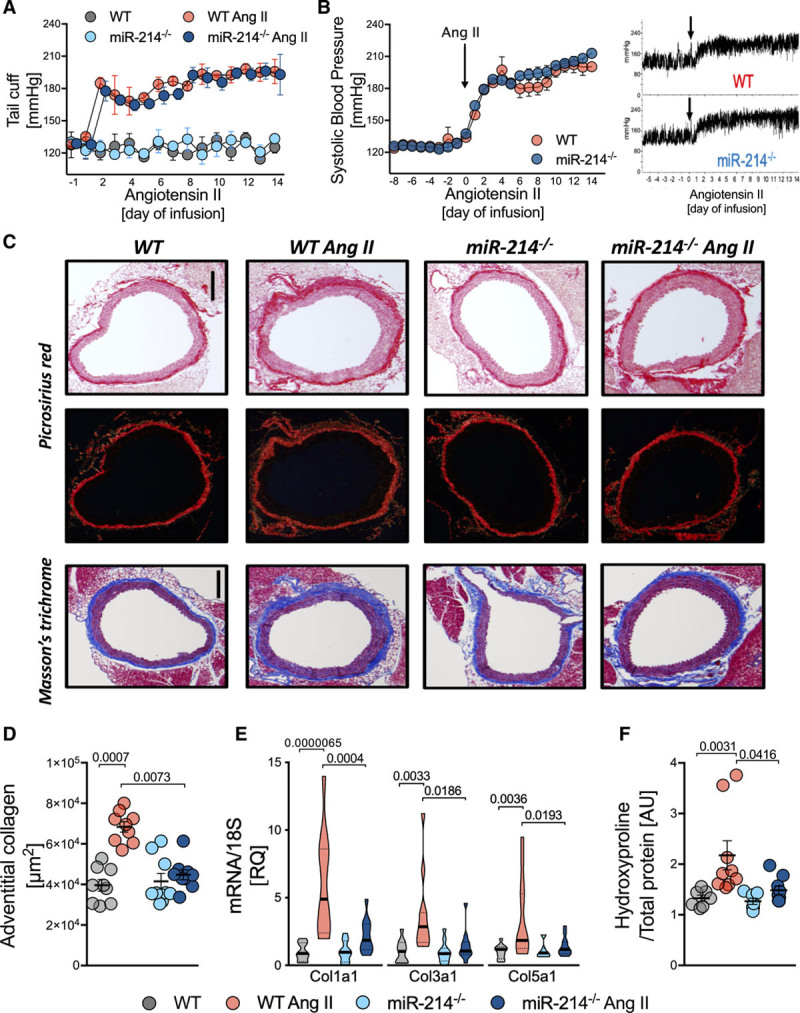
**Pivotal role of miR-214 in the regulation of perivascular fibrosis in Ang II (angiotensin II)-dependent hypertension.** **A**, Tail-cuff BP in sham and Ang II infused miR-214^−/−^ and WT littermates (n=9/group). **B**, Systolic BP measured by telemetry at baseline and during Ang II infusion (average, left; example readings, right; n=5). **C**, Perivascular collagen accumulation in picrosirius red (top) and Masson trichrome (bottom) staining (representative of n=9; scale bar=300 *μ*m). **D**, Quantitative analysis of perivascular collagen deposition in Masson trichrome (n=9/group). **E**, Collagen 1, 3, and 5 mRNA in miR-214^−/−^ and WT littermates infused with buffer or Ang II (n=11/group). **F**, Aortic collagen quantification by hydroxyproline assay (n=7 in Sham and n=9 in Ang II). Data presented as mean±SEM; repeated measures 2-way ANOVA (**A** and **B**), Kruskal-Wallis test with FDR correction (**D** and **F**; *P* values adjusted for 6 comparisons) or 2-way ANOVA with Tukey test (**E**; *P* values adjusted for 6 comparisons). Overall *P* values for repeated measures 2-way ANOVA; **A** (*P*^Time^=0.0051, *P*^Group^=1.8×10^−8^, *P*^Time×Group^=0.1378); **B** (*P*^Group^=0.06, *P*^Time^=2.6×10^−7^, *P*^Group×Time^=0.83); for 2-way ANOVA; **E**, for Col1a1 (*P*^AngII×Genotype^=0.003, *P*^AngII^=1.3×10^−5^, *P*^Genotype^=0.0035), Col3a1 (*P*^AngII×Genotype^=0.037, *P*^AngII^=0.0034, *P*^Genotype^=0.033) Col5a1 (*P*^AngII×Genotype^=0.028, *P*^AngII^=0.0053, *P*^Genotype^=0.042), for Kruskal-Wallis; **D** (*P*=0.0003) and **F** (*P*=0.0006).

To understand the functional role of miR-214 in hypertension, we used miR-214^−/−^ mice in which we investigated development of hypertension, as well as vascular dysfunction and remodeling. In vivo, miR-214^−/−^ mice did not show altered blood pressures either at baseline or throughout 14-day Ang II infusion evaluated by both tail cuff (Figure 2A) and telemetry (Figure 2B). Importantly, in spite of that, we observed a significant reduction in periaortic collagen accumulation/fibrosis in miR-214^−/−^ mice using Picrosirius red and Masson’s trichrome staining (Figure 2C and 2D). In wild-type mice, aortic *Col1a1*, *Col3a1*, and *Col5a1* were all significantly induced by Ang II, but this increase was abolished in miR-214^−/−^ mice (Figure 2E). Furthermore, hydroxyproline accumulation was observed in the aortas of Ang II infused WT but not miR-214^−/−^ mice (Figure 2F).

**Figure 3.**
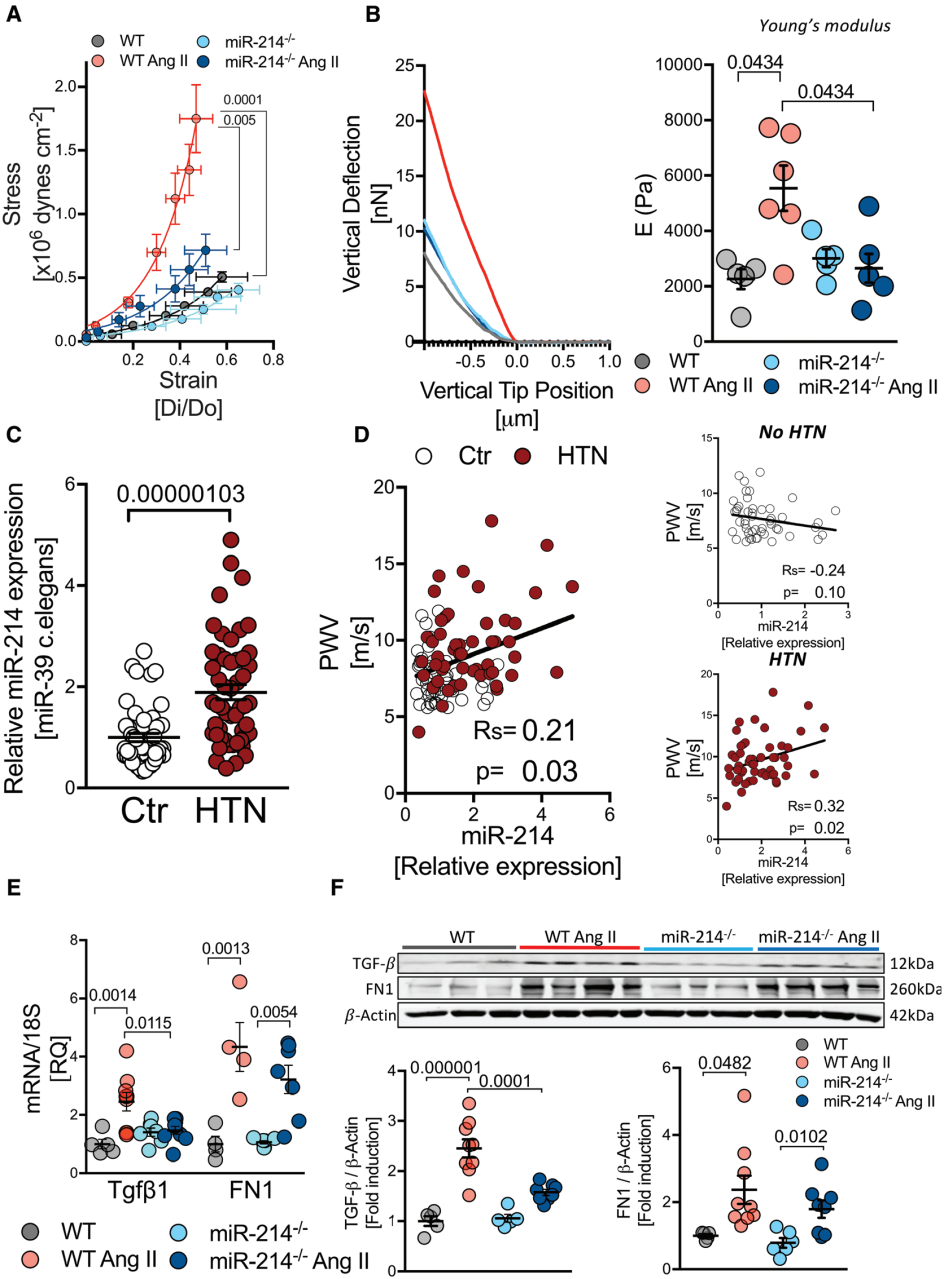
**Role of miR-214 in the regulation of vascular stiffening in mouse and human hypertension.** **A**, Thoracic aorta stiffness studied by pressure myography (n=5–8). **B**, Elastic modulus of adventitial stiffness measured by atomic force microscopy (right) with representative force-indentation curves (left; n=5–6/group). **C**, Circulating miR-214 serum levels in normotensive (Ctr, n=49) and patients with hypertension (HTN, n=51). **D**, Correlation between plasma miR-214 levels and pulse wave velocity (PWV) in humans with and without hypertension (n=100). **E**, TGF-β (transforming growth factor β) and fibronectin mRNA expression in control and hypertensive miR-214^−/−^ and WT mice (n=4–9/group). **F**, Protein level of TGF-β and fibronectin in aortas studied by WB (n=5–9). Data presented as mean±SEM repeated measures 2-way ANOVA with Bonferroni correction (**A**), Kruskal-Wallis test with FDR correction (**B**; *P*-adjusted for 6 comparisons) or 2-way ANOVA with Tukey test (**E** and **F**; *P* values adjusted for 6 comparisons) or *t*-test (**C**). Spearman rank test was used to assess correlations (**D**). Overall *P* values for 2-way ANOVA; **A** (*P*^AngII^=1.8×10^−5^, *P*^Genotype^=0.042), *P*^AngII×Genotype^=0.097), **E** for Tgfb1 (*P*^AngII×Genotype^=0.0059, *P*^AngII^=0.0031, *P*^Genotype^=0.225), FN1 (*P*^AngII^=1.2×10^−^^5^, *P*^Genotype^=0.266, *P*^AngII×Genotype^=0.218); **F** for TGF-β (*P*^AngII×Genotype^=0.0034, *P*^AngII^=5.1×10^−7^, *P*^Genotype^=0.0087), for FN1 (*P*^AngII^=0.0015, *P*^Genotype^=0.243, *P*^AngII×Genotype^=0.589) and Kruskal Wallis **B** (*P*=0.0368).

Functional consequences of perivascular fibrosis were studied using assessment of vascular stiffness. MiR-214^−/−^ mice were protected from the development of hypertensive vascular stiffening in pressure myography in mouse aortas including adventitial layer (Figure 3A). Nanoindentation studies utilizing atomic force microscopy also showed an increase of Young’s modulus of the aortic wall in wild-type mice with Ang II infusion, while this increase was abolished in miR-214^−/−^ mice (Figure 3B).

### miR-214 Is Associated With Vascular Stiffness in Human Hypertension

To assess translational relevance of these observations to human hypertension, we investigated plasma miR-214 levels in patients with and without hypertension (for detailed clinical characteristics, please see Online Table I), and in relation to PWV, as a measure of vascular stiffness.^7^ MiR-214 levels were higher in patients with hypertension (Figure 3C). Notably, there was a significant correlation between plasma miR-214 levels and PWV (Figure 3D). This relationship remained significant in multiple regression analysis after correcting for blood pressure and age (miR-214/PWV: β*=0.212; *P*=0.02). No significant relationship between miR-214 and age was identified by multiple regression.

### miR-214 Modulates Vascular TGF-β in Ang II Hypertension

To further understand the role of miR-214 in the regulation of perivascular fibrosis, we studied the expression of *Tgfb1* (TGF-β) and fibronectin (FN1). While both TGF-β and FN1 were induced in Ang II infused WT mice, only TGF-β mRNA and protein induction were abolished in the aorta of miR-214^−/−^ mice (Figure 3E and 3F), indicating indirect regulation of TGF-β by miR-214.

### miR-214 Regulates Vascular Dysfunction and Perivascular Inflammation in Hypertension Independently of Blood Pressure

**Figure 4.**
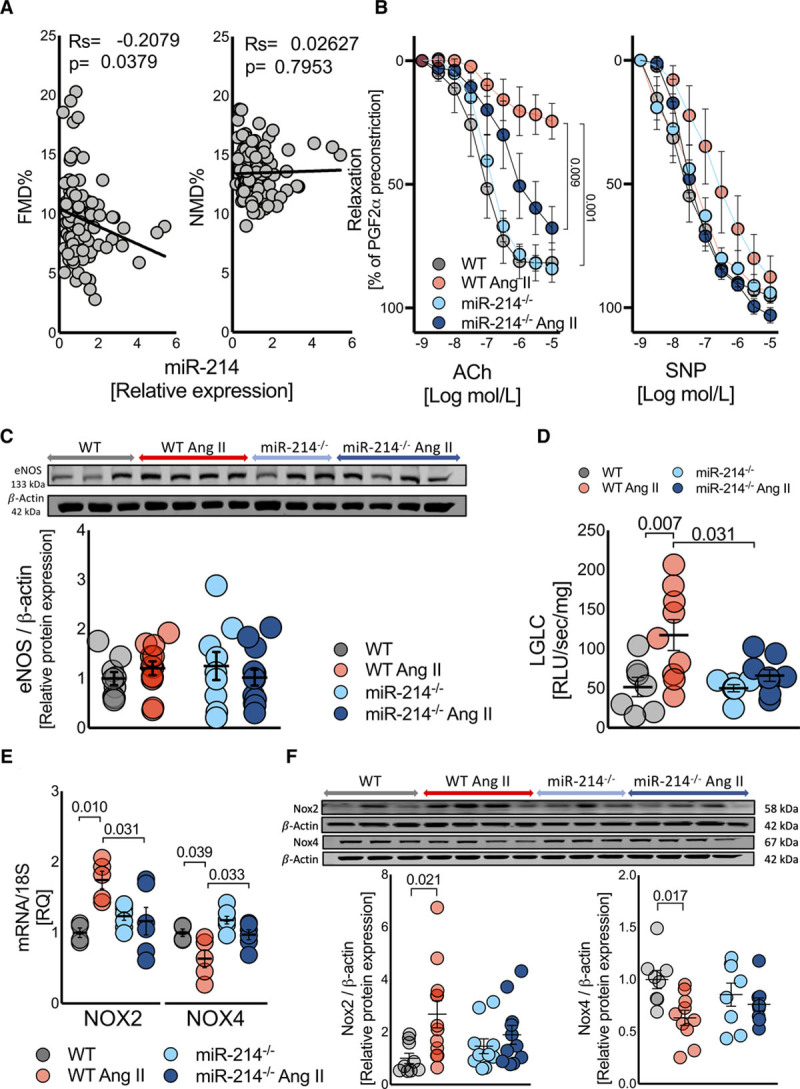
**Essential role of miR-214 in endothelial dysfunction and vascular oxidative stress in hypertension.** **A**, Relationship between plasma miR-214 levels and endothelial function (flow mediated dilatation [FMD]) or nonendothelium-dependent relaxations to nitroglycerin (NMD) in humans (n=100). **B**, Isometric tension studies of endothelium dependent (acetylcholine; ACh) and independent (sodium nitroprusside [SNP]) vasorelaxations (n=5–7/group) using wire myography in sham buffer and Ang II (angiotensin II) infused WT and miR-214^−/−^ mice. **C**, eNOS (endothelial nitric oxide synthase) protein level in mouse aortas (Western blotting; n=9–12/group). **D**, Aortic superoxide production measured by lucigenin (5 μM) enhanced chemiluminescence (n=7–9/group). **E**, Aortic Nox2 and Nox4 mRNA expression (n=5/group) and (**F**) protein levels in aortas of sham and Ang II infused miR-214^−/−^ and WT mice (n=9–12/group). Data presented as mean±SEM and analyzed by Spearman rank correlation test (**A**), repeated measures 2-way ANOVA with Bonferroni correction (**B**) or 2-way ANOVA with Tukey post hoc test (**C–F**; *P* values adjusted for 6 comparisons). Overall *P* values for repeated measures 2-way ANOVA; **B** for Ach (*P*^Response^=0.0009, *P*^Group^=0.044, *P*^Response×^^Group^=0.016) and SNP (*P*^Response^=04.1×10^−^^7^, *P*^Group^=0.049, *P*^Response×Group^=0.217). Two-way ANOVA; **C** (*P*^AngII^=0.949, *P*^Genotype^=0.862, *P*^AngII×Genotype^=0.235); **D** (*P*^AngII×Genotype^=0.061, *P*^AngII^=0.0042, *P*^Genotype^=0.051); **E** for Nox2 (*P*^AngII×Genotype^=0.0062, *P*^AngII^=0.193, *P*^Genotype^=0.203) and Nox4 (*P*^AngII×Genotype^=0.331, *P*^AngII^=0.002, *P*^Genotype^=0.005); **F** for Nox2 (*P*^AngII^=0.0096, *P*^Genotype^=0.683, *P*^AngII×Genotype^=0.116) and Nox4 (*P*^AngII^=0.0093, *P*^Genotype^=0.924, *P*^AngII×Genotype^=0.109).

**Figure 5.**
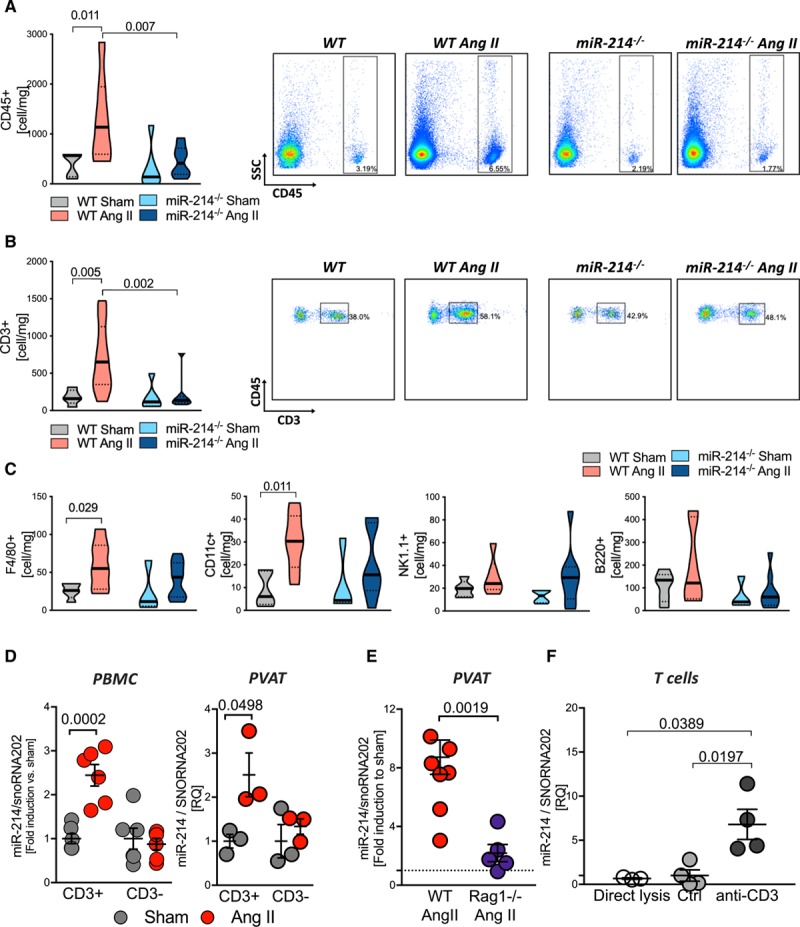
**Crucial role of miR-214 in regulation of perivascular inflammation in hypertension.** **A**, Total number of leukocytes and T cells (**B**) with representative density plots (n=6–9/group). **C**, Number of perivascular macrophages (F4/80+), dendritic cells (CD11c+), B cells (B220+), and NK cells (NK1.1+) per mg of tissue, studied by flow cytometry (n=5–7/group). Data presented on volcano plots depicting median (^**__**^) and quartiles (^….^). **D**, miR-214 induction in T cells (CD3+) and remaining leukocytes (CD3−) in Ang II hypertension measured in cells from peripheral blood (left, PBMC n=5–6/group) and from perivascular adipose tissue (PVAT; right, n=3/group of 3 pulled mice). **E**, MiR-214 level in PVAT of WT and RAG1^−/−^ animals (n=5–7/group). **F**, miR-214 in quiescent (direct lysis; control mAb) and anti-CD3 mAb activated T cells (n=3–4/group). Data presented as mean±SEM and analyzed by Kruskal-Wallis test (**A–C**), 2-way ANOVA with Tukey post hoc test (**D**; *P* values adjusted for 6 comparisons), *t*-test (**E**), and by Kruskal-Wallis test with FDR (**F**; *P* values adjusted for 3 comparisons). Overall *P* values for Kruskal-Wallis; **A** (*P*=0.017), **B** (*P*=0.011), **C** (*P*=0.06), **D** (*P*=0.05), and **F** (0.0001); 2-way ANOVA; **D** for PBMC (*P*^CD3×AngII^=0.0006, *P*^AngII^=0.0026, *P*^CD3^=0.0006) and PVAT (*P*^AngII^=0.024, *P*^CD3^=0.115, *P*^CD3×AngII^=0.115).

A strong link between miR-214 and vascular phenotype was further supported by correlations of plasma miR-214 with NO-dependent endothelial vascular function, measured by flow-mediated dilatation, but not with the endothelium-independent responses to nitroglycerine (NMD; Figure 4A). These observations in humans were corroborated in miR-214^−/−^ mice, which were protected from the development of endothelial dysfunction, demonstrating preserved NO-mediated vasodilatation, in spite of Ang II-induced hypertension (Figure 4B). No differences in nonendothelium-dependent vasorelaxations to sodium nitroprusside were observed (Figure 4B). As eNOS (endothelial nitric oxide synthase) is a predicted miR-214 target, changes of eNOS expression could explain alterations of endothelial function. However, in aortas of Ang II-treated mice, we did not observe changes in total eNOS protein, compared with miR-214^−/−^ mice (Figure 4C). As oxidative stress is known to mediate both endothelial dysfunction and vascular stiffness in hypertension, we next investigated vascular superoxide production and Nox2 and Nox4 nicotinamide adenine dinucleotide phosphate oxidases. MiR-214^−/−^ mice showed reduced induction of vascular oxidative stress upon Ang II infusion, as measured by superoxide production (Figure 4D) and *Nox2* nicotinamide adenine dinucleotide phosphate oxidase mRNA and protein level (Figure 4E and 4F). These changes in vascular function and oxidative stress were accompanied by reduced perivascular recruitment of total leukocytes (Figure 5A) and CD3+ T cells (Figure 5B) in miR-214^−/−^ hypertensive animals in comparison to hypertensive WT mice. Additionally, Ang II infusion significantly elevated the total number of macrophages and dendritic cells (CD11c+CD11b−) only in WT animals, but not in miR-214^−/−^ mice (Figure 5C). No significant changes were observed with respect to B cells or NK cells (Figure 5C).

### miR-214 Induction in the T Cells Is Essential for Perivascular Fibrosis

To further understand the mechanisms of the effects of miR-214 on vascular inflammation in hypertension, we next focused on the effects of hypertension on immune cell miR-214. In leukocytes isolated from both peripheral blood mononuclear cells and PVAT, we observed miR-214 induction only within CD3+ cells (T cells), and not in CD3 negative cells (Figure 5D). Furthermore, miR-214 did not increase in PVAT of RAG-1^−/−^ mice, lacking mature T cells, despite Ang II infusion (Figure 5E). This further supports the hypothesis that infiltrating T cells are an essential source of the increase of miR-214 observed in PVAT.

In keeping with the role of blood pressure elevation in miR-214 induction, miR-214 was not induced in T cells isolated from mice subjected to blood pressure-lowering with hydralazine and hydrochlorothiazide (Online Figure III). Moreover, we found that 24-hour stimulation of T cells using anti-CD3 antibodies elevated miR-214 levels in vitro (Figure 5F). In contrast, stimulation of mouse primary fibroblasts and of primary vascular smooth muscle cells, endothelial cells, or THP-1 monocytes in culture using Ang II or cytokine combinations did not significantly alter miR-214 expression level (Online Figure IV).

**Figure 6.**
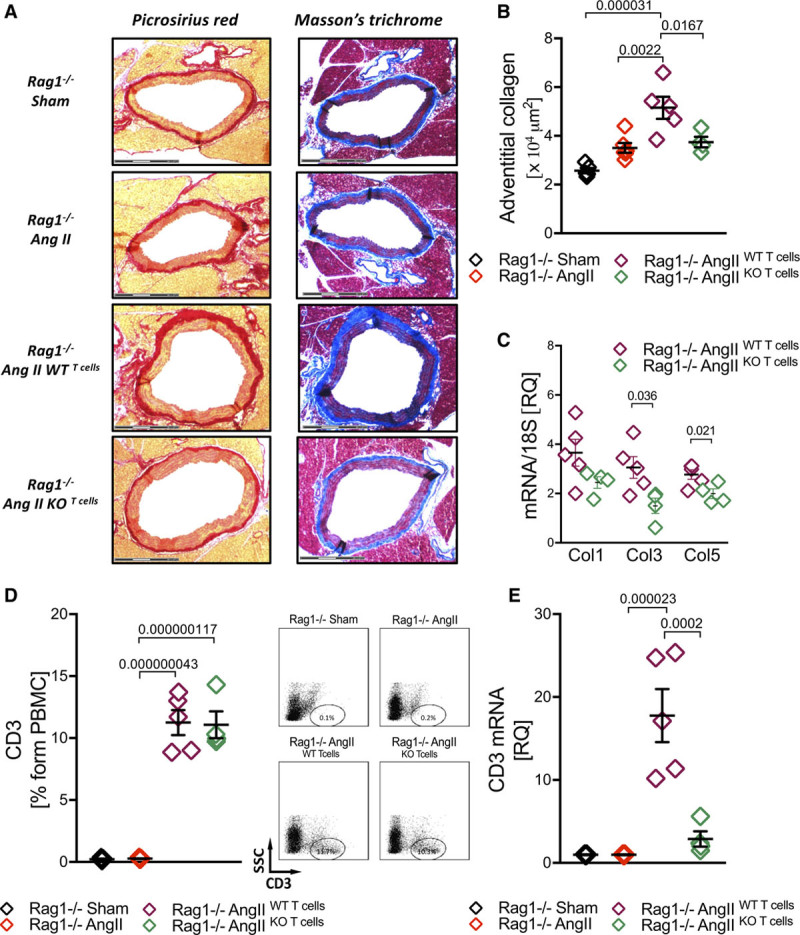
**T cell miR-214 mediates perivascular fibrosis and profibrotic T cell accumulation in PVAT (perivascular adipose tissue) in Ang II (angiotensin II)- induced hypertension.** **A**, Vascular collagen accumulation in response to Ang II-hypertension assessed using picrosirius red (left) and Massons trichrome (right; scale bar=300 µm, representative of n=4–5/group) with quantification of adventitial collagen (**B**) in RAG1^−/−^ mice and upon adoptive transfer of WT and miR-214^−/−^ T cells (n=4–5/group). **C**, Ang II-dependent induction of Col 1, 3, and 5 mRNA in aortas of RAG1^−/−^ transferred with WT or miR-214^−/−^ T cells (n=4–5). **D**, Levels of circulating T cells in RAG1^−/−^ mice and upon adoptive transfer of WT or miR-214^−/−^ T cells (left) with representative dot plots (right; n=4–5/group). **E**, CD3 mRNA in PVAT of RAG1^−/−^ mice and upon adoptive transfer of WT or miR-214^−/−^ T cells (n=4–5/group). Data presented as mean±SEM and analyzed by 1-way ANOVA with Tukey post hoc *t* test (**B**, **D**, **E**; *P* values adjusted for 6 comparisons), *t*-test with FDR (**C**; *P* values adjusted for 3 comparisons). Overall *P* values for 1-way ANOVA; **B** (*P*=6.3×10^−5^), **D** (*P*=1.5×10^−8^), and **E** (*P*=7.8×10^−6^).

To understand the functional role of T cell miR-214 in hypertensive vascular remodeling, we performed adoptive transfer of either wild type or miR-214^−/−^ T cells to RAG1^−/−^ mice and studied perivascular fibrosis and collagen accumulation. We confirmed earlier findings of Wu et al^11^ that RAG1^−/−^ mice are protected from development of perivascular fibrosis and it is rescued by adoptive transfer of wild type T cells (Figure 6A and 6B). This effect was observed only when wild type, but not when miR-214^−/−^ T cells were transferred (Figure 6A and 6B), while level of hypertension was similar irrespective of T cell miR-214 status. This was accompanied by reduced aortic collagen expression in RAG1^−/−^ transferred with miR-214^−/−^ T cell when compared with WT T cells (Figure 6C). Similar levels of peripherally reconstituted circulating T cells were found in RAG1^−/−^ mice upon adoptive transfer of WT and miR-214^−/−^ cells (Figure 6D). At the same time, we found more than a 5-fold reduction of *cd3* mRNA in the PVAT after transfer of miR-214^−/−^ T cells in comparison to WT T cell transfer (Figure 6E).

### miR-214 and Mineralocorticoid Signaling

As mineralocorticoid signaling is important in modulation of both fibrosis and T cell function in hypertension,^22^ we have investigated potential changes of mineralocorticoid signaling in T cells in miR-214^−/−^ mice. Plasma aldosterone levels were unaltered in miR-214^−/−^ mice (Online Figure VA). Furthermore, upon Ang II infusion, there were no changes in either mineralocorticoid (MR) or glucocorticoid receptor (GR) mRNA or protein level in T cells (Online Figure VB and VC). We also analyzed the effects of aldosterone on anti-CD3 dependent T cell fibrosis-related cytokine mRNA in vitro in WT and miR-214^−/−^ T cells. Aldosterone induced only TGF-β mRNA in WT T cells, while other studied cytokines were not significantly induced. This induction was not observed in miR-214^−/−^ mice (Online Figure VD). These studies show that MR signaling is unlikely to a play critical role in the effects of miR-214 on perivascular fibrosis.

### Transcriptional Profiling Reveals Crucial Role of miR-214 in Hypertensive T Cell Activation

**Figure 7.**
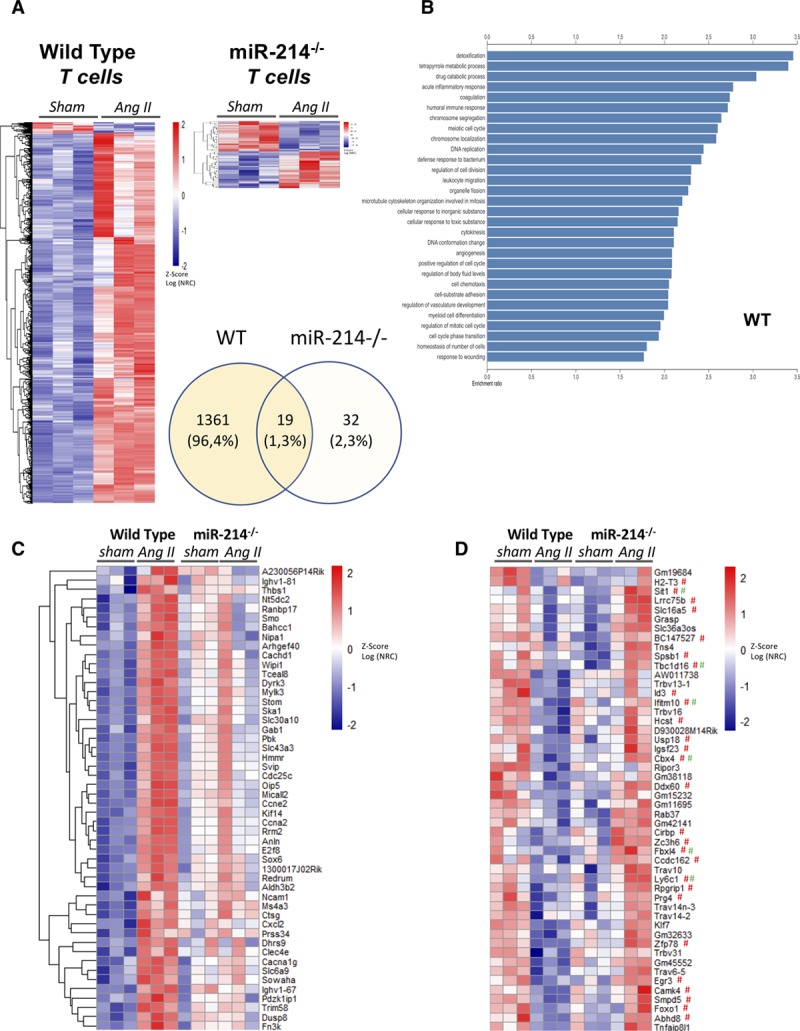
**Essential role of miR-214 in T cell responses in Ang II (angiotensin II)-induced hypertension.** **A**, Genes with absolute log_2_FC>1, significantly (FDR adj *P*<0.05 calculated using Wald test in DESeq2) changed in T cells following 2-week AngII infusion in vivo in WT (left) and mir214^−/−^ (right) mice (n=3). Venn diagram indicates total numbers of genes changed in each strain of mice in response to Ang II. **B**, Top 30 pathways significantly upregulated in WT T cells following 2-weeks AngII infusion in vivo in WT mice. **C**, Top 50 genes (based on interaction term estimate) preferentially induced by Ang II infusion in WT when compared with mir214^−/−^ mice (FDR adj *P*_interaction_ <0.05 for all presented). **D**, Top 50 genes preferentially repressed by AngII infusion in WT as compared with miR-214^−/−^ mice. Predicted targets of miR-214 for miRWalk (# red) and TargetScan 7.1 (# green). Data on heat maps are presented as *z*-scores of log transformed, normalized gene read counts (Log NRC) calculated with DESeq2.

To understand how miR-214 in the T cell could modulate the profibrotic T cell phenotype in hypertension, we performed RNASeq analysis of isolated splenic T cells from sham and Ang II infused WT and miR-214^−/−^ mice. This analysis has shown that in vivo Ang II infusion induced at least a 2-fold change in 1380 genes in WT T cells, while miR-214^−/−^ T cells remained greatly unresponsive with only 51 genes altered (Figure 7A). Relevant gene sets significantly affected by Ang II hypertension in WT T cells included cytokine metabolic processes, leukocyte migration, leukocyte cell-cell adhesion, and the regulation of T cell activation. None of these were changed in miR-214^−/−^ (Figure 7B). Only 19 of these genes overlapped between WT and miR-214^−/−^ further emphasizing the extent of the reprogramming of hypertensive T cells by miR-214. This is also exemplified by diversity of genes which are significantly induced in WT but not in miR-214^−/−^ T cells (Figure 7C; analysis using interaction term with FDR correction). Conversely, a large number of the top 50 genes significantly repressed in WT T cells, but not in miR-214^−/−^ T cells were the predicted targets for this miR (Figure 7D), further supporting a pleiotropic role of miR-214 in transcriptional reprograming of T cells by Ang II.

### miR-214 Regulates T Cell Activation and Chemotaxis in Hypertension

**Figure 8.**
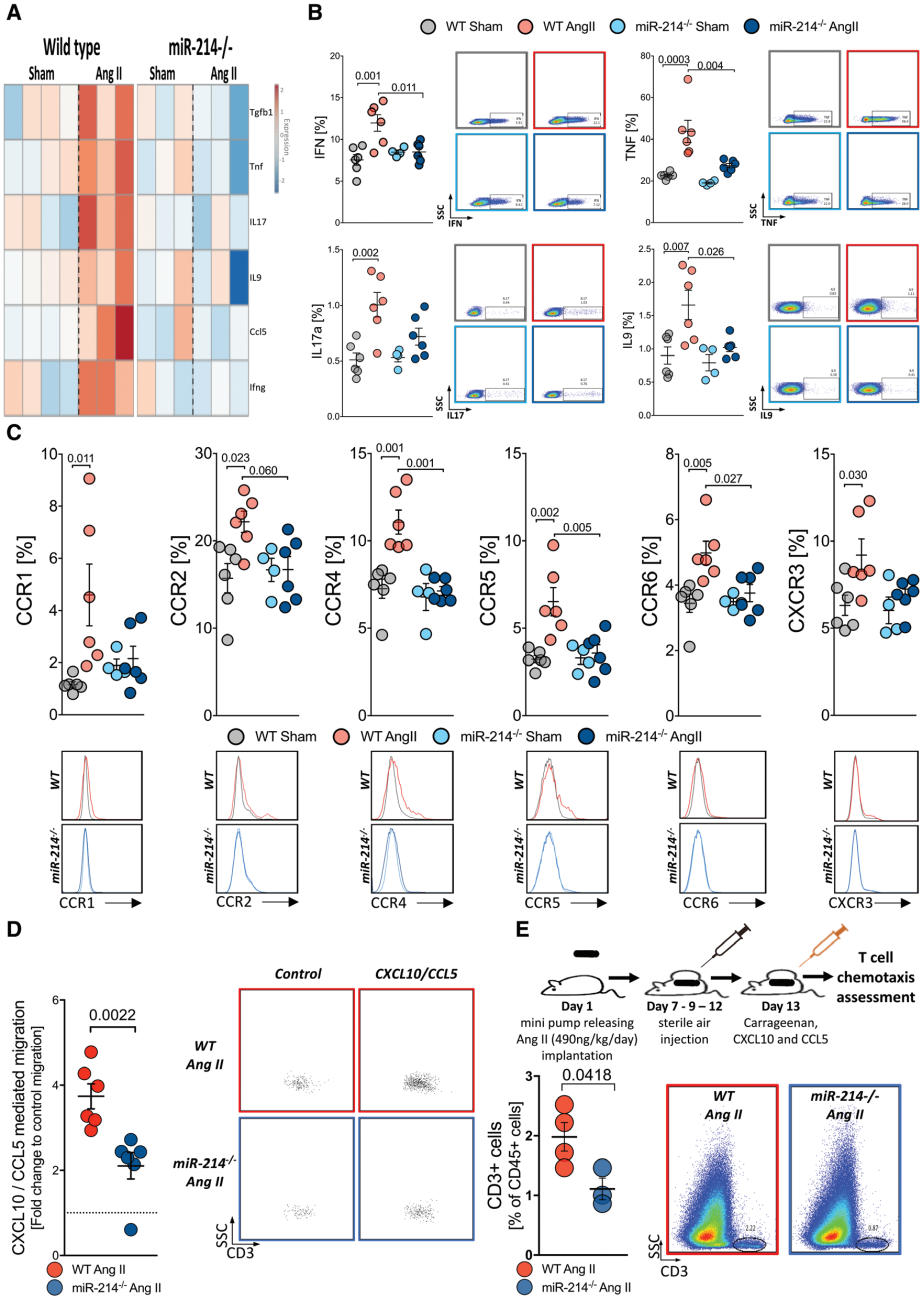
**MiR-214 modulates T cell proinflammatory cytokine production, chemokine receptors expression and T cells migration in Ang II (angiotensin II)-induced hypertension.** **A**, Heatmap of selected profibrotic cytokine mRNA in T cells upon Ang II-induced hypertension in WT (left) and miR-214^−/−^ (right) mice. Gene expression was studied upon ex vivo activation by anti-CD3 and anti-CD28 for 24 h (n=3–4/group) and presented as fold change, relative to average of sham. **B**, Cytokine production by T cells in sham buffer and Ang II infused WT and miR-214^−/−^ mice (n=4–6/group) upon 6-h stimulation with PMA studied by flow cytometry. **C**, T cell chemokine surface receptors in sham and Ang II-infused WT and miR-214^−/−^ mice studied by flow cytometry with representative histograms (n=4–6/group). **D**, In vitro chemotaxis of T cells obtained from hypertensive animals toward CXCL10 and CCL5 in Boyden chambers (n=6/group) with representative dot plots reflecting control and chemokine induced migration. **E**, Air pouch model of T cell chemotaxis (top) showing T cell recruitment toward membranes surrounding air-pouch filled with carrageenan and recombinant proteins (CXCL10 and CCL5) in hypertensive WT and miR-214^−/−^ mice with representative density plots (n=3–4/group). Data presented as mean±SEM and analyzed by *t*-test with FDR (All genes show FDR adj *P*<0.05 in WT but not in miR-214^−/−^), Mann-Whitney *U* test (**D**) or *t*-test (**E**). Two-way ANOVA with Tukey (**B** and **C**; *P* values adjusted for 6 comparisons). Overall *P* values for 2-way ANOVA; **B** for IFN (*P*^AngII×Genotype^=0.0073, *P*^AngII^=0.0068, *P*^Genotype^=0.0988), TNF (*P*^AngII×Genotype^=0.0475, *P*^AngII^=0.0001, *P*^Genotype^=0.0042), IL17a (*P*^AngII^=0.0007, *P*^Genotype^=0.1316, *P*^AngII×Genotype^=0.0814) and IL9 (*P*^AngII^=0.0045, *P*^Genotype^=0.0245, *P*^AngII×Genotype^=0.0994); **C** for CCR1 (*P*^AngII×Genotype^=0.0413, *P*^AngII^=0.0198, *P*^Genotype^=0.2508), CCR2 (*P*^AngII×Genotype^=0.0467, *P*^AngII^=0.0452, *P*^Genotype^=0.1193), CCR4 (*P*^AngII×Genotype^=0.0081, *P*^AngII^=0.0022, *P*^Genotype^=0.0013), CCR5 (*P*^AngII× Genotype^=0.0142, *P*^AngII^=0.0047, *P*^Genotype^=0.0186), CCR6 (*P*^AngII×Genotype^=0.0445, *P*^AngII^=0.0066, *P*^Genotype^=0.0596), CXCR3 (*P*^AngII^=0.0167, *P*^Genotype^=0.0771, *P*^AngII×Genotype^=0.163).

Following the analysis of our RNASeq data, we next studied mRNA and protein levels of key proinflammatory and profibrotic cytokines upon Ang II-induced hypertension. mRNA expression was studied in activated T cells isolated from spleens of sham and Ang-II infused WT or miR-214^−/−^ mice. As expected, Ang II hypertension caused increased induction of *Il17a, Tgfb1 Tnfa, Il9, Ccl5, and Ifng* in the WT T cells. MiR-214^−/−^ mice were protected from this induction (Figure 8A). The effects of miR-214 on the induction of T cell protein levels of IL-17, TNF-α (tumor necrosis factor-α), Il-9, and IFN-γ (interferon-γ) in vivo were confirmed by intracellular staining using flow cytometry (Figure 8B).

Chemokine-dependent perivascular recruitment of T cells is essential for vascular dysfunction^19^ and aortic stiffening.^11^ To further investigate this phenomenon, we studied expression of relevant chemokine receptors in normotensive and hypertensive WT and miR-214^−/−^ mice. We observed induction of CCR1, CCR2, CCR4, CCR5, and CCR6 as well as CXCR3 on the surface of T cells in Ang II hypertension (Figure 8C). These increases were not observed in miR-214^−/−^ mice (Figure 7C), suggesting that reduced chemotaxis of these cells can explain decreased T cell recruitment in miR-214^−/−^ mice. Therefore, we next investigated the role of miR-214 in the regulation of T cell chemotaxis in vitro using the Boyden chamber and in vivo air-pouch experiments (Figure 8D and 8E). Peripheral blood T cells from the miR-214^−/−^ mice infused with Ang II exhibited reduced chemotaxis towards key chemokines involved in hypertension—CCL5 (C-C motif chemokine ligand 5) and CXCL10 when compared with WT mice (Figure 8D). This observation was confirmed using an in vivo chemotaxis model, in which T cell recruitment into the air-pouch wall was inhibited by miR-214^−/−^ in Ang II-hypertensive mice (Figure 8E).

## Discussion

We have identified the role of miR-214 in mediating perivascular fibrosis in hypertension. We link these pathophysiological effects of elevated miR-214 to the T cell compartment that infiltrates the PVAT and induces the fibrotic response. MiR-214 affects global change of transcriptomic profile in T cells in hypertension, enabling their activation and recruitment into perivascular fat. It also regulates the development of T cell profibrotic properties in hypertension. These mechanistic studies in mice led us to show in humans that plasma miR-214 is increased in hypertension and is significantly associated with PWV and endothelial dysfunction in hypertensive subjects. Hence, miR-214 is a new target linking the T-cell inflammatory cascade to vascular complications of hypertension.

While our study is the first to link miR-214 to vascular dysfunction and perivascular fibrosis, earlier studies have shown that miR-214 regulates cardiac and renal fibrosis in the conditions of organ-selective stress. MiR-214 repression is protective in the setting of unilateral ureteral obstruction-induced renal fibrosis and damage, but at the same time miR-214 loss exacerbates cardiac fibrosis evoked by ischemia-reperfusion injury.^13^ Those studies indeed highlight the organ-specific context in which miRNAs can drive opposing responses, presumably due to the different cell types, in which the miRNA is expressed in and the cell-specific transcriptome induced in response to different pathogenic stimuli.

We report that miR-214^−/−^ mice are protected from the development of endothelial dysfunction, a hallmark of hypertension,^23^ despite the similar blood pressure response to Ang II. While this might be regulated by the role of miR-214 in the endothelium itself, we have previously shown that T cell infiltration into the vessel wall induces endothelial dysfunction mainly through IFN-γ-dependent mechanisms.^19^ In line with these observations, miR-214^−/−^ prevented T cell activation and IFN-γ release.

The mechanisms through which T cells and their cytokines such as IL-17, IFN-γ, TNF-α, and TGF-β can affect perivascular fibrosis have been previously demonstrated.^24–28^ In our model, TGF-β seems to be the most evident regulator of fibrosis in aortas from Ang II hypertensive mice. Its induction is alleviated in miR-214^−/−^ mice, even though TGF-β is not a direct target of miR-214. This effect may however be explained by the fact that TGF-β expression in fibroblasts may be induced by T-cell–derived cytokines, such as IFN-γ, TNF-α, or IL-17.^24^

Induction of miR-214 in PVAT and T cells is dependent on blood pressure increases, rather than by direct actions of Ang II. This is in line with earlier observations that T cell activation and vascular recruitment depend on pressure increase and are in part dependent on mechanical stretch,^11^ which involves and affects antigen presenting cell activation.^29^ Taking into account that miR-214 increases in T cells upon activation and perivascular T cell infiltration increases 3- to 4-fold in hypertension, T cell infiltration into PVAT may provide the most compelling mechanism of miR-214 increase in this compartment in Ang II-induced hypertension. Moreover, miR-214 seems to regulate T cell activation characterized by profibrotic effector cytokine production and chemokine receptor expression. The mechanisms of this are complex and not fully understood. RNASeq analysis of isolated T cells showed that Ang II causes profound transcriptional changes, which are lost in miR-214^−/−^ T cells. It is difficult to molecularly dissect a single repressor of T cell activation and migration that could be responsible for the effects of miR-214. RNASeq suggested that miR-214 in the T cells has a broad pleotropic effect, leading to global change in T cell activation in hypertension. The combined effect of these alterations is likely responsible for the beneficial phenotypic changes in hypertension. Further studies are needed to fully understand all the pathways central to these effects orchestrating vascular inflammation and stiffness and to develop possible selective therapeutic targeting approaches.

While our study shows the role of miR-214 in T cell activation and cytokine production in hypertension for the first time, one should be cautious in attempts to apply these findings to other inflammatory conditions. For example, miR-214 decreases in relapsing phase of multiple sclerosis, which is accompanied by increased Th17 cells.^18^

Interestingly, we observe that the induction of major hypertensive cytokines is inhibited in T cells from Ang II-infused miR-214^−/−^ mice, but no effects on blood pressure responses was seen in this model. This is surprising but may reflect a complex role of cytokines at different stages of hypertension, as well as a possible differential role of vascular and renal inflammation at different stages of hypertension. In fact, our blood pressure data are in contrast to very recent suggestions that intrarenal application of antagomir targeting miR-214-3p significantly attenuated salt-induced hypertension and albuminuria in SS rats. miR-214-3p directly targeted eNOS in Dahl salt-sensitive rats.^16^ However, in our model, we did not see decreases in eNOS expression in the vessels upon Ang II infusion. This discrepancy may be related to different model of hypertension used. Congenic Dahl salt-sensitive rat used in that study is primarily kidney-dependent,^30^ which may contribute to observed difference. Interdependence with other immune cells involved in hypertension, such as B cells,^31^ macrophages,^32^ and NK cells^33^ should be considered as well. While our studies do not show miR-214 induction in non-T cell immune cells, their role may be upstream from the T cell in immune activation.^33^

Finally, it is important to note that we reported that miR-214 is increased in plasma of patients with hypertension and is linked to PWV as a measure of vascular stiffening in humans.^34^ Thus, miR-214 might serve as a valuable biomarker of vascular stiffening. Moreover, considering the pivotal role of miR-214 in regulating vascular inflammation, endothelial dysfunction, oxidative stress, and perivascular fibrosis, all constituting elements of accelerated vascular aging, our study indicates a possibility of the use of miR-214 as a biomarker of accelerated vascular aging and in the future targeting T cell miR-214 to target this important process. Our studies also emphasize the need to identify novel therapies inhibiting T cell activation and their recruitment in cardiovascular diseases.^35,36^

In summary, we have identified that miR-214 is an essential regulator of T cell dependent mechanisms of vascular stiffening, fibrosis, and dysfunction in hypertension. It may represent a novel biomarker and a possible target for modulation of perivascular inflammation.

## Acknowledgments

We thank Dr Eric Olson for providing the miR-214^−/−^ mice and Dr John McClure for valuable statistical support.

## Sources of Funding

This study was supported by European Research Council (TJG-InflammaTENSION: ERC-CoG-726318 and AHB–Vascmir: ERC-338991), National Science Centre, Poland (2011/03/B/NZ4/02454), British Heart Foundation (Guzik-FS/14/49/30838; Baker-RG/14/3/30706, CH/11/2/28733) and Kidney Research UK (LD–PD6/2012), MRC (MC, MR/S005412/1), UK Regenerative Medicine Platform (MSS, MR/R015651/1), National Science Centre, Poland (RN funded by 2013/11/N/NZ4/00310).

## Disclosures

None.

## Supplemental Materials

Expanded Materials and Methods

Online Figures I–VI

Raw gene counts of the RNA-Seq

Full Unedited Blots

Major Resources Table

References^37–43^

## Supplementary Material



## Novelty and Significance

### What Is Known?

- Hypertensive vascular stiffening, characterized by perivascular fibrosis, is a determinant of accelerated vascular aging and predictor of cardiovascular morbidity and mortality.
- Perivascular fibrosis is mediated by immune cell infiltration and linked to Th17 lymphocytes.
- MiR-214 is important in regulating cardiac and renal fibrosis and is expressed by immune cells.
