# Supplementary material for: T-Cell–Derived miRNA-214 Mediates Perivascular Fibrosis in Hypertension
Source: Circ Res. 2020 Feb 17;126(8):988–1003. doi: 10.1161/CIRCRESAHA.119.315428 (PMC7147427; doi:10.1161/CIRCRESAHA.119.315428)
Supplement: Supplementary file 1 [file res-126-988-s001.pdf]

Supplemental Material

T cell-derived miRNA-214 controls perivascular fibrosis in hypertension.

Ryszard Nosalski[1,2], Mateusz Siedlinski[2], Laura Denby[3], Eilidh McGinnigle[1], Michal Nowak[2], Aurelie Nguyen Dinh Cat[1], Laura Medina-Ruiz[4], Marco Cantini[5], Dominik Skiba[1,2], Grzegorz Wilk[2], Grzegorz Osmenda[2], Julie Rodor[3], Manuel Salmeron-Sanchez[5], Gerard Graham[4], Pasquale Maffia[1,4,6], Delyth Graham[1], Andrew H. Baker[3] and Tomasz J. Guzik[1,2]

[1]Institute of Cardiovascular and Medical Sciences, University of Glasgow, UK.

[2]Department of Medicine, Jagiellonian University Medical College, Krakow, Poland.

[3]Centre for Cardiovascular Science, Queen's Medical Research Institute, University of Edinburgh, UK

[4]Institute of Infection, Immunity and Inflammation, University of Glasgow, UK.

[5]Centre for the Cellular Microenvironment, School of Engineering, University of Glasgow, UK

[6]Department of Pharmacy, University of Naples Federico II, Naples, Italy.

DETAILED MATERIALS AND METHODS:

Materials: Please see the Major Resources Table in the Supplemental Materials.

Animals and model of hypertension

C57BL/6 and Rag1$^{-/-}$ (B6.129S7-*Rag1*^{tm1Mom}/J) were obtained from Jackson Laboratories. The miR-214$^{-/-}$ mice were kindly gifted by Dr Eric Olson previously described[15], which for adoptive transfer experiments were backcrossed 7 times to C57BL/6N background.

Hypertension was induced in twelve-week old mice by infusion of angiotensin II (490ng/kg/d) for 14 days via osmotic minipumps (Model 2002, Alzet Corporation). Sham treatment involved infusion of the vehicle for angiotensin II. In the subset of animals hydralazine (320mg/L) and hydrochlorothiazide (60mg/L) was co-administrated ad libitum in drinking water to normalise blood pressure as described before[11]. Mice received hydralazine for two weeks starting 1 day before osmotic minipumps implantation. Blood pressure (BP) was measured by non-invasive tail cuff plethysmography using BP2000 Blood Pressure Analysis System (Visitech Systems Inc.), following a 7 days period of training prior to minipumps implantation. BP was monitored every day at the same time with the platform warmed to 36°C. 10 preliminary and 15 actual measurements in each session were performed. Animals were rotated through all channels daily to avoid channel-to-channel variations. The Chauvenet's criterion (measurements greater than 2 SD form the mean) was used to automatically detect and exclude the outliers by the BP-200 Software.

Independent group of animals were used for telemetric BP assessment. A telemetric device (PA-C10, Data Sciences International; DSI) was implanted into mice through catherization of carotid artery one week prior to minipump implantation. BP was recorded for 10 minutes each hour for the whole duration of the experiment.

All surgical procedures were performed under general anaesthesia by using isoflurane (3-5% for induction and 1,5-3% for anaesthetic maintenance) or ketamine with xylazine solution IP (100 mg/kg and 10 mg/kg). At the end of experiments animals were sacrificed with CO_2 inhalation.

Randomization and allocation concealment were performed. Briefly, mice were randomly allocated to treatment groups, sham mice were implanted minipumps containing buffer, and were labelled using animal facility labelling system. Operators for all assays were blinded to group allocation during all analytical procedures.

All *in vivo* work was performed in accordance with the United Kingdom Animals Scientific Procedures Act 1986 and ARRIVE (Animal Research: Reporting of In Vivo Experiments) Guidelines and approved by the Home Office under Project License No. 7009021 and animal ethics approval of Jagiellonian University Ethics Committee (157/2016). All experiments conform the guidelines from Directive 2010/63/EU of the European Parliament on the protection of animals used for scientific purposes.

T cell adoptive transfer

T cells were isolated by negative selection from the spleen of miR-214$^{-/-}$ or wild-type littermate animals according to the manufacturer's instructions using EasySep™ Mouse T cell Isolation Kit (STEMCELL Technologies). 10^7 cells were resuspended in 200ul of saline and injected by tail vein into RAG1$^{-/-}$ animals. 4 weeks after adoptive transfer chronic angiotensin II infusion was infused as above.

Vascular studies

Vessel segments from thoracic aorta were mounted in organ bath chambers (610M myograph, DMT) filled with PSS buffer (118,99 mmol/l NaCl, 4.69 mmol/l KCl, 1.17 mmol/l $MgSO_4$-$7H_2O$, 1,18 mmol/l KH_2PO_4, 2,5 mmol/l $CaCl_2$-$2H_2O$, 25 mmol/l $NaHCO_3$, 11.1 mmol/l glucose, 0,03 mmol/l EDTA) oxygenated with 95%O_2-5%CO_2). Relaxation to the endothelium-dependent and -independent vasodilators, acetylcholine (ACh) and sodium nitropruside (SNP), following preconstruction with prostaglandin F2α ($PGF_{2\alpha}$) was measured. All relaxation responses are presented as a percentage of initial constriction to $PGF_{2\alpha}$.

Vascular stiffness was assessed using pressure myograph system (DMT) as previously described[37]. Briefly, thoracic aorta was mounted on cannulas in calcium-free PSS buffer and intraluminal pressure was increased in step wise-fashion between 10 and 120 mmHg, preceded by 60 min equilibration period at pressure 70 mmHg in 37°C. Internal and external diameters were recorded.

Superoxide generation was measured in intact thoracic aorta vessels using lucigenin enhanced chemiluminescence [LGLC]. Harvested blood vessels were cut to expose endothelial surface and placed in Krebs HEPES buffer (37°C) containing 5μM lucigenin into single tube FB12 luminometer (Berthold). Data are expressed as relative light units (RLU) per second per mg of a dry vessel tissue.

Histologic Stains

Formalin fixed and paraffin embedded 7μm sections of thoracic aorta with PVAT were deparaffinized and rehydrated. For picrosirius red staining, sections were stained with Weigert's iron hematoxylin solution (Sigma-Aldrich) for 10 minutes. Next slides were washed and incubated in the dark with 0.1% Sirius red F3B (Sigma-Aldrich) for 1 hour, washed in acidified water, dehydrated and mounted. Masson's trichrome staining was performed in accordance with the manufacturer's protocol using HT15 Trichrome stain Kit (Sigma-Aldrich). Quantification of collagen staining was performed using ImageJ software by blinded observers.

Hydroxyproline assay

Snap frozen thoracic aortas were hydrolyzed in 6N HCl at 120°C for 24h and neutralized with 6N NaOH. From each sample, 5 ul of the final neutralized hydrolysate was used.

Hydroxyproline concentration was measured using a microplate reader and normalized to the protein concentration.

Flow cytometry

Perivascular adipose tissue was separated firm the aorta and digested using collagenase type IX (125U/ml), collagenase type I (450 U/ml) and hyaluronidase (60 U/ml) dissolved in PBS containing calcium, magnesium and 20μM HEPES for 20 minutes in 37°C. The digested tissue was passed through a 70μm sterile cell strainer to yield a single cells suspension. Cells were washed and resuspended in FACS buffer, counted and stained using monoclonal antibodies and live/dead marker. The antibodies and their clones for staining were as follows: CD45 (30-F11, #103108), CD3 (145-2C11, #100320), CD11b (M1/70, #101208), F4/80 (BM8, #123130), CD11c (N418, #117316), B220 (RA3-6B2, #103212), NK1.1 (PK136, #108726) and zombie aqua viability dye. Example of gating strategy used to identify leukocyte subsets is shown in Online Figure VI.

Spleens were collected and passed through 70μm sterile cell strainer to obtain a single cell suspension. Next spleenocytes were stained for CD3 (145-2C11, #100312) and chemokine receptors such as CCR2 (SA203G11, #150608), CCR5 (HM-CCR5, eBioscience, #12-1951-83), CCR1 (S15040E, #152503), CXCR3 (CXCR3-173, #126542), CCR6 (29-2L17, #129815) and CCR4 (2G12, #131204).

To evaluate cytokine production by T cells intracellular staining was performed. Isolated spleenocytes were stimulated with phorbol 12-myristate13-acetate (PMA) and ionomycin for 6 hours in the presence of Golgi stop (eBioscience) in RPMI 1640 supplemented in 10% FBS. After washing cells were stained for CD3 (145-2C11, #100312) and subsequently fixed, permeabilized using Foxp3/transcription factor staining buffer set (eBioscience) and stained for intracellular cytokines for 30 min in 4°C IFN (XMG1.2, #505813), TNF-α (MP6-XT22, BD Bioscience, #554419), IL-17a (TC11-18H10.1, #506904), IL-9 (RM9A4, #514111). All antibodies were obtained from BioLegend, unless otherwise stated. Stained cells were acquired using FACS LSRII (BD Bioscience) and analyzed using FlowJo software (Tree Star Inc.).

MicroRNA profiling

Total RNA from 5-6 pools of two PVAT (surrounding aorta) each, was isolated using mirVana kit (Ambion) and treated with rDNAse I. RNA was reverse transcribed using Megaplex primmer pools and profiled with TaqMan Rodent MicroRNA A Array v.2.0 (Applied Biosystems). Ct values were generated using automatic threshold and baseline settings in RQ Manager software (ver.1.2.2, Applied Biosystems). Ct values (<35) were normalized using global mean method and compared between treatment groups using t-test. All statistical tests were performed on normalized Ct values in R (ver. 2.15.0) using HTqPCR (ver.1.10.0) package.

Analysis of miRNA and Gene Expression

RNA and miRNA were isolated using miRNeasy mini kit (Qiagen) and reverse transcription was performed using High Capacity cDNA reverse kit (Applied Biosystems) or TaqMan MicroRNA reverse transcription kit with the RT specific primers converting miRNA (Applied Biosystems). MiRNeasy serum/plasma kit (Qiagen) was used for isolation of miRs form human plasma in accordance with the manufacturer's instructions. During the serum miRNA purification 3.5 ul of miRNeasy/Serum/Plasma Spike-In Control ($1.6x10^8$ copies/ul of *C. elegans* miR-39, Qiagen) was added for use as an internal control. Reverse transcription was performed using TaqMan miRNA Reverse transcription kit (ThermoFisher). Measurement of miRNA and gene expression mRNA level were performed using specific TaqMan probes (Applied Biosystems) on the 7900HT real-time PCR system (ThermoFisher) according with manufacturer’s instructions. SNORNA202 or U6 were used as an endogenous control for miRNA, miR-39 *C. elegans* was used as an endogenous control for serum miRNA level while 18S rRNA was used as endogenous control for gene expression. Relative quantification was calculated as $2^{-\Delta\Delta Ct}$.

In Situ Hybridization

In situ hybridisation was performed to visually localise the miR-214 as previously described[38]. Briefly, 5μm tissue sectionNs were rehydrated and treated with 20μg/ml proteinase K (#25530049, Life Technologies) at 37 degrees for 20 minutes, then fixed with 4% paraformaldehyde. After washing with PBS, slides were incubated with hybridization buffer at 60 or 58 degrees for miR-214-3p (#YD00611471) or scramble probe (#YD00699004) labeled with 3′ and 5′ digoxigenin (DIG, Exiqon). After washing and blocking, immunodetection was performed with an anti-DIG antibody (#11093274910, Roche) in concentration 1:500 overnight. MiR-214 was visualized with BM purple solution (#11442074001, Roche) after 5 hours at room temperature.

Cell Culture

Primary fibroblasts and vascular smooth muscle cells were obtained from adult C57BL/6 animals. Aortas were cleaned from periaortic fat, adventitia was separated mechanically from

media and next obtained layers were placed separately into enzyme solution (Collagenase II, Elastase, Soybean Trypsin Inhibitor in HBSS with calcium and magnesium) for 1 hour at 37°C in 5% CO_2 in the incubator. Cells were cultured in DMEM (Gibco) supplemented with 10%FBS until 90% confluent.

THP-1 monocytes (American Type Culture Collection) were cultured in RPMI 1640 medium supplemented with 10% FCS in 37°C. Next THP-1 monocytes were treated with 100nM phorbol 12-myristate 13-acetate (PMA) for 48h in order to differentiation into non-polarized macrophages (M0). Subsequently, M0 macrophages were cultured for 48h in presence of 20ng/ml IFN-γ (PeproTech) and 10ng/ml LPS (Sigma-Aldrich) or IL-4 20 ng/ml (PeproTech) and IL-13 20ng/ml (PeproTech) to differentiate into M1 or M2 phenotype, respectively.

Human microvascular endothelial cells (HMVEC, ThermoFisher Scientific) were cultured in Medium 131 containing Microvascular Growth Supplement (MVSG) and gentamicin/amphotericin solution (Gibco). Primary VSMCs, fibroblasts and HMVEC were stimulated with angiotensin II (Sigma-Aldrich), IL-17, TNFα, TGFβ or ET-1 (R&D Systems) for 24 or 48 hours. Cells from 4-5 passage were used for experiments. Mouse T cells obtained from splenocytes of C57BL/6 mouse (negative selection) were stimulated via anti-CD3 antibodies (clone 17A2, BioLegend) with or without aldosterone (1μM) for 24 hours in RPMI 1640 supplemented in 10% FBS.

Pulse Wave Velocity (PWV) and Flow Mediated Dilatation (FMD)

Arterial stiffness in human subjects with and without hypertension (see Table S1 for detailed clinical characteristics) was assessed by pulse wave analysis using a validated system (Sphygmocor Mx, AtCor Medical) at the time of recruitment. Measurement was performed accordingly to manufacturer instruction and in accordance with guidelines published by Laurent et al[7] and analyzed using SphygmoCor CvMS ver.9. PWV (Pulse Wave Velocity) was calculated as the distance covered by the waves (in meters) divided by the time delay measured between the feet of the two waveforms (in seconds). All patients underwent 24 hours blood pressure monitoring using Spacelabs 90217 Ultralite BP Monitors. Plasma was collected from the patients for laboratory risk biomarkers and microRNA detection and frozen immediately in -80°C. Flow-mediated dilatation (FMD) of the brachial artery was used to determine the vascular endothelial function and nitroglycerine-mediated dilatation (NMD) was used for measuring endothelial-independent vasodilatation. FMD was performed before and after five-minute long brachial artery occlusion while NMD one, two and five minutes after sublingual nitroglycerin application. Analysis was performed using Vascular Tools 5 software. Study was performed according to the Declaration of Helsinki and was approved by the Ethics Committee of Jagiellonian University. All participants gave written informed consent.

Western blot

Immunoblotting was used to examine the expression of Nox2 (BD, #611414), Nox4 (Novus, #NB110-58849) eNOS (BD, #610297) TGF-β (Cell Signaling, #37115) FN1 (Sigma, #F3648), MRC (Santa Cruz Biotechnology, #sc-71554) , GR (Santa Cruz Biotechnology , #sc-1004) and beta-actin (Abcam, #ab8227).

Snap frozen vessels or isolated T cells were homogenized in RIPA lysis and extraction buffer (Thermo Scientific) supplemented with Halt™ Protease and Phosphatase Inhibitor Cocktail (Thermo Scientific). Protein were separated by electrophoresis on 10% or a gradient SDS polyacrylamide gels and transferred to nitrocellulose membrane. Nonspecific binding sites were blocked with 5% skim milk or 5% BSA in Tris-buffered saline solution containing 0.1% Teween-20 (TBS-T) for 1 hour in room temperature. Membranes were incubated with specific antibodies overnight at 4°C (5% BSA TBS-T), washed three times and incubated with secondary antibodies conjugated with fluorescent dyes (IRDye®800CW or IRDye®680LT,

LI-COR) for 1 hour in room temperature. Signals were detected using Odyssey CLx Imaging System (LI-COR) and quantified with ImageStudio software (LI-CORE).

Aldosterone Assay

Plasma, obtained from blood of heparinized animals, was centrifuged immediately after collection (2000 x g for 15 minutes at 4$_{o}$C), aliquoted and stored in -80$_{o}$C. Before the analysis plasma samples were diluted 3 times in calibration diluent RD5-69. Aldosterone level was determined using Aldosterone Assay (KGE016, R&D) in accordance with the manufacturer's instructions.

Air-pouch model of inflammation

The air-pouch model of T cell recruitment was utilized as described previously[39] with some modifications. In brief, hypertension was induced in twelve-week old mice by infusion of angiotensin II for 14 days as described above. 7 days upon the start of Ang II infusion, 3 ml of sterile air were injected subcutaneously under the dorsal skin every 2 days on 3 occasions. 24 hours after the final air injection, inflammation was induced by injecting 1ml of sterile carrageenan (1% (w/v) in PBS, Sigma) and 100 µl of PBS containing 1µg of recombinant proteins (CXCL10 and CCL5, PeproTech) into the air-pouch. 48 hours later, mice were culled and the membrane surrounding the air-pouch was dissected and digested in 1ml of HBSS containing 0.44 Wunsch units of Liberase (Roche) for 1 hour at 37$_{o}$C. Liberase was then deactivated with 20 µL of FBS and cell suspensions were passed through 70 µm nylon mesh filters and washed. Single cell suspensions were then stained with viability dye (eFluor$_{TM}$ 506, eBioscience, #65-0866-14) and monoclonal antibodies (CD45 PerCP Cy5.5 #103132 and CD3 APC #100312, BioLegend) and next acquired using an LSRFortessa flow cytometer (BD Bioscience). Analysis was conducted using FlowJo software (Tree Star Inc.).

Chemotaxis of isolated T cells

Chemotaxis was examined on isolated T cells in Boyden chambers with 5 µm filter pore sizes (Costar). 600 µl of medium (RPMI 1640 supplemented in 10% FBS) containing recombinant chemokines (CXCL10 and CCL5) or vehicle was pipetted into the bottom chamber. Top insert containing 2x10$_{5}$ of T cells in 100 µl of RPMI 1640 supplemented in 10% FBS (medium) was carefully placed on top of the plate and incubated in humified chamber in 5% CO_2 at 37$_{o}$C for 1 hour. Following this, the cells were collected from the bottom chamber, centrifuged and next resuspend in 100 µl of FASC buffer. Cell were analyzed using flow cytometry. Data presented as a fold change of chemokine induced migration versus native migration.

Measurement of the elastic modulus of the adventitia via atomic force spectroscopy

Atomic force spectroscopy was employed to measure the mechanical properties of explanted mouse aortas using a Nanowizard 3 BioScience AFM (JPK, Germany) as described previously[40]. 10 µm silica microspheres (Corpuscular Inc, USA) were glued to tipless cantilevers (TL-CONT from NanoWorld AG, Switzerland) using a two-component epoxy glue (Araldite from Huntsman Advanced Materials GmbH, Switzerland) and employed as nanoindenters. Explanted mouse aortas were embedded in OCT (Tissue-Tek®), frozen in liquid nitrogen and cut into 10 µm thick sections using a cryostat. Sections were rehydrated in DPBS (Gibco, USA) to remove the OCT prior to the measurement. Measurements were carried out in DPBS at RT. Cantilevers were calibrated in DPBS against a rigid surface to determine sensitivity using a contact-based method and the thermal noise method was employed to determine the spring constant using the JPK SPM Desktop software (version 6.1.130). 4x4 µm$_{2}$, 4x4 pixels maps were measured in four positions in the adventitia of each animal. Indentation curves were obtained by applying a maximum force of 30 nN, with a retraction

length of 10 µm and a constant speed of 2 µm s^{-1}, and were analysed using the Hertz model to an indentation depth of 1 µm and with Poisson's ratio of 0.5 using the JPK Data Processing software (version 6.1.120).

RNA-Seq Analysis

Total RNA from T cells was isolated using Direct-zol™ RNA MiniPrep kit (Zymo Research) and treated with DNase I. RNA QC was performed using Agilent 2100 Bioanalyzer System. Samples with RIN score between 9.7-10 were used for further sequencing as described previously[41]. Briefly, mRNA was first enriched with Oligod(T) beads. Enriched mRNAs were fragmented for 15 minutes at 94 °C. First strand and second strand cDNA were subsequently synthesized. cDNA fragments were end repaired and adenylated at 3'ends, and universal adapters were ligated to cDNA fragments, followed by index addition and library enrichment by PCR with limited cycles. The sequencing library was validated on the Agilent TapeStation (Agilent Technologies, Palo Alto, CA, USA), and quantified by using Qubit 2.0 Fluorometer (Invitrogen, Carlsbad, CA) as well as by quantitative PCR (KAPA Biosystems, Wilmington, MA, USA). Sequencing was performed with HiSeq 4000 (Illumina) with 150 bp paired-end reads (PE150). Sequence reads were trimmed to remove possible adapter sequences and nucleotides with poor quality using Trimmomatic v.0.36. The trimmed reads were mapped to the Mus musculus GRCm38 reference genome available on ENSEMBL using the STAR aligner v.2.5.2b. Unique gene hit counts were calculated by using featureCounts from the Subread package v.1.5.2. The hit counts were summarized and reported using the gene_id feature in the annotation file. Only unique reads that fell within exon regions were counted. After extraction of gene hit counts, the gene hit counts table was used for downstream differential expression analysis (DESeq2).

Statistical analysis

Data are reported as the mean±standard mean error. To test normality of distribution, Kolmogorov-Smirnov, Shapiro-Wilk, q-q-plots and histograms test were employed before parametric or non-parametric tests were applied. Correlations were assessed by Spearman's rank correlation analysis. Statistical analysis was performed using t-test (2-group studies), one-way or two-way ANOVA (>2-group studies) with a Tukey's or FDR multiple comparison *post hoc* test for normally distributed data and Mann-Whitney or Kruskal-Wallis test with FDR correction for non-normally distributed data using GraphPad Prism 7 or Statistica 13 TIBCO. P values <0.05 were consider significant. Reported p values represent values already after multiple comparison correction (Bonferroni, Tukey's or FDR). Analysis in patient populations were performed in Statistica 13 TIBCO using t test for independent samples. Correlation between PWV and plasma miR-214 was assessed by Spearman's rank correlation test and then by multiple regression correcting for SBP and age.

Differential gene expression analysis from the RNA-Seq experiment was performed using raw gene counts and DESeq2 package in R (ver. 3.5.1)[42]. Primary analysis employed Wald test and investigated effect of Ang II infusion on transcriptome profile in WT (n=3 for Sham and n=3 for Ang II groups) and miR-214$^{-/-}$ (n=3 for Sham and n=3 for AngII groups) T cells separately. Secondary analysis was performed using both WT and miR-214$^{-/-}$ samples and employed Wald test in order to identify genes with significant interaction term between Ang II and genotype, while adjusting for these two independent factors. Overrepresentation analysis was performed using WebGestalt[43] using significantly (FDR adj p<0.05) changed genes.
Number of samples represent biological replicates (independent mice used for experiments).

Online Table I. Clinical characteristics of patients studied.

**Clinical features**	**Control (n=49)**	**Hypertension (n=51)**	**p value**
Age (years ± SD)	55.5±7	58.0±7	NS
Male sex (n; %)	22 (45%)	28 (54%)	NS
**Blood pressure characteristics**			
Hypertension (%)	0%	100%	<0.001
Office blood pressure	120±20/77±8	141±17/87±11	<0.001
24h ABPM (systolic mmHg ± SD)	115±8	130±15	<0.001
24h ABPM (diastolic mmHg ± SD)	73±6	79±10	<0.001
Serum creatinine (μmol/L)	89±11	87±12	NS
**Cardiovascular risk characteristics**			
Current Smoking (%)	10 (20%)	11 (21%)	NS
Ever smoking status(%)	13 (27%)	14 (27%)	NS
Glucose intolerance/DM	2 (4%)	6 (11%)	NS
Current blood glucose (mmol/L)	5.1±0.8	5.7±1.5	<0.05
Total cholesterol (mmol/L)	6.1±1.2	5.8±1	NS
**Inflammatory/Autoimmune disease**	0 (0%)	0 (0%)	NS
CRP level	3.8±11	2.9±3.6	NS
WBC (cells/mm3)	6.1±2	6.9±4	NS
**Atherosclerosis and CAD**			
PCI/CABG(%)	0 (0%)	0 (0%)	NS
Prior myocardial infarction (%)	0 (0%)	0 (0%)	NS
Peripheral Arterial Disease (%)	0 (0%)	1 (2%)	NS
Stroke/TIA	0 (0%)	1 (2%)	NS
**Medications**			
ACE inhibitors/ ARB (%)	0 (0%)	32 (63%)	<0.05
Beta – blockers (%)	0 (0%)	20 (51%)	<0.05
Calcium channel blockers (%)	2 (4%)	11 (70%)	<0.05
Alpha – blockers (%)	0 (0%)	2 (4%)	NS
Diuretics (%)	0 (0%)	18 (35%)	<0.05
Statins (%)	2 (4%)	11 (22%)	<0.05

Online Figure I

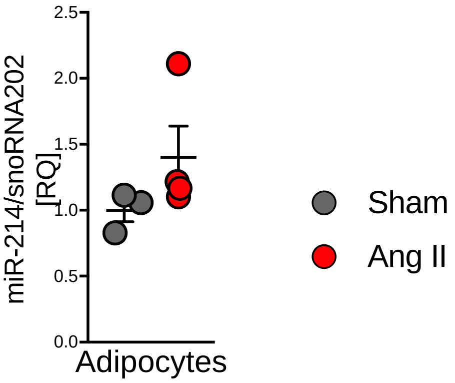

Online Figure I. **miR-214 level in adipocytes** Level of miR-214 in adipocytes isolated from PVAT of sham and Ang II animals. Data presented as mean±SEM and analyzed by Mann-Whitney U test (n=3-4, two animals pulled in each - 6-8 mice).

Online Figure II

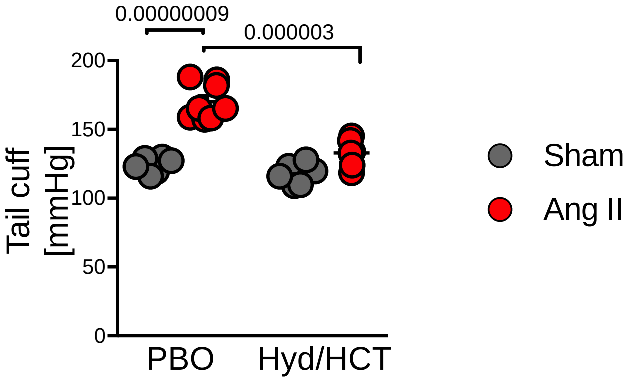

Online Figure II. **Effect of antihypertensive treatment on blood pressure in Sham and Ang II animals** Systolic blood pressure of mice treated with placebo (PBO) or hydralazine/hydrocholrothiazide (Hyd/HCT) measured by tail cuff. Data presented as mean±SEM (n=6-8/group) and analyzed using two-way ANOVA ($p_{Drug \times AngII}$=6.4e-04, p_{Drug}=1.2e-05, p_{AngII}=8.3e-08) with Tukey's multiple comparison test (p-values adjusted for 6 comparisons).

Online Figure III

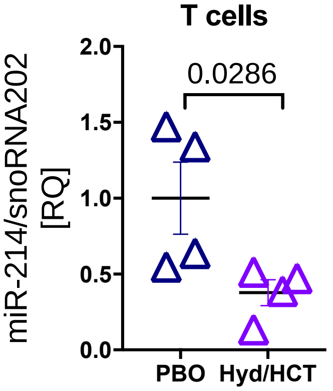

Online Figure III. **Antihypertensive treatment prevents miR-214 elevation in T cells** MiR-214 level in splenic T cells isolated form placebo (PBO) or Hydralazine/Hydrocholothiazide (Hyd/HCT) treated hypertensive (Ang II) mice (n=4/group). Data presented as mean±SEM and analyzed by Mann-Whitney U test.

Online Figure IV

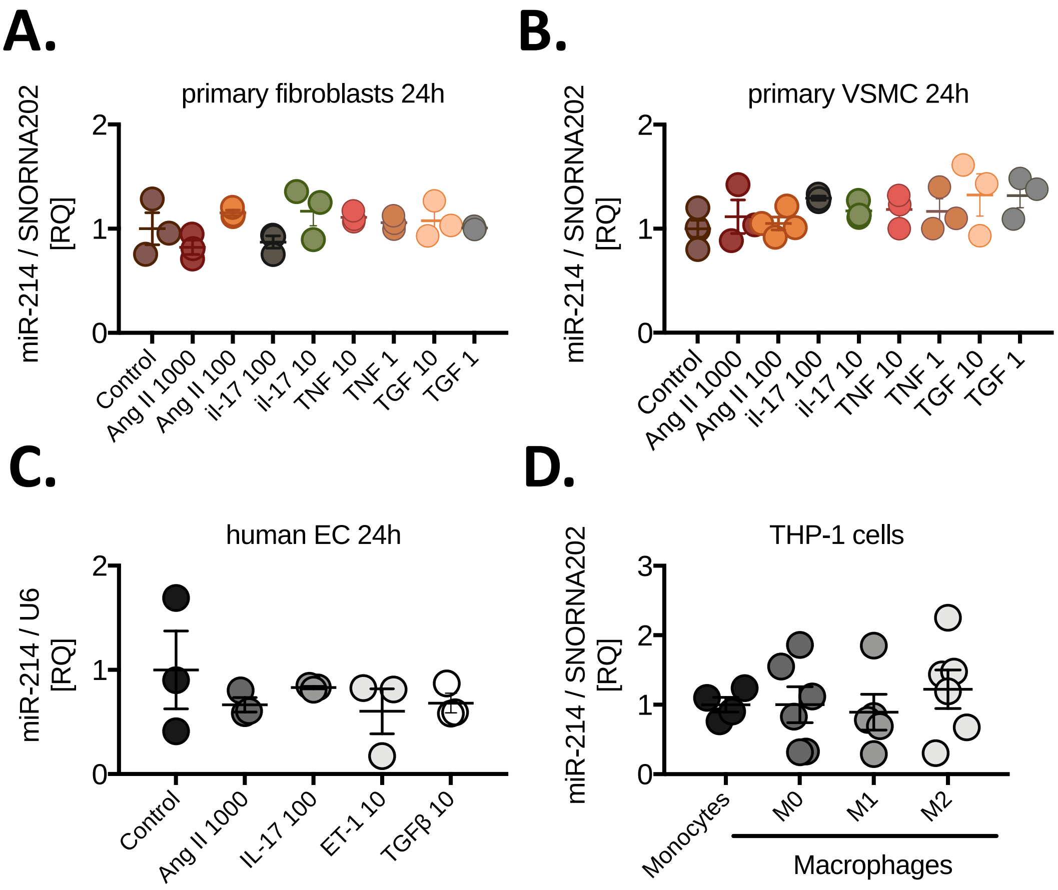

Online Figure IV. **miR-214 mRNA in vascular cell types *in vitro* upon stimulation** Level of miR-214 in primary mouse fibroblasts (**A.**) and primary mouse vascular smooth muscle cells (**B.**) upon stimulation by Ang II, IL-17, TNF-alpha and TGF-β for 24h. **C**. miR-214 expression in human endothelial cells upon stimulation of Ang II, IL-17 and TGF-beta for 24h. **D**. miR-214 expression in THP-1 cells. Data presented as mean±SEM (n=3-6). Data analyzed using one-way ANOVA. Overall one-way ANOVA analysis for Panel **A**; p=0.11, Panel **B**; p=0.35, Panel **C**; p=0.64 and Panel **D**; p=0.81.

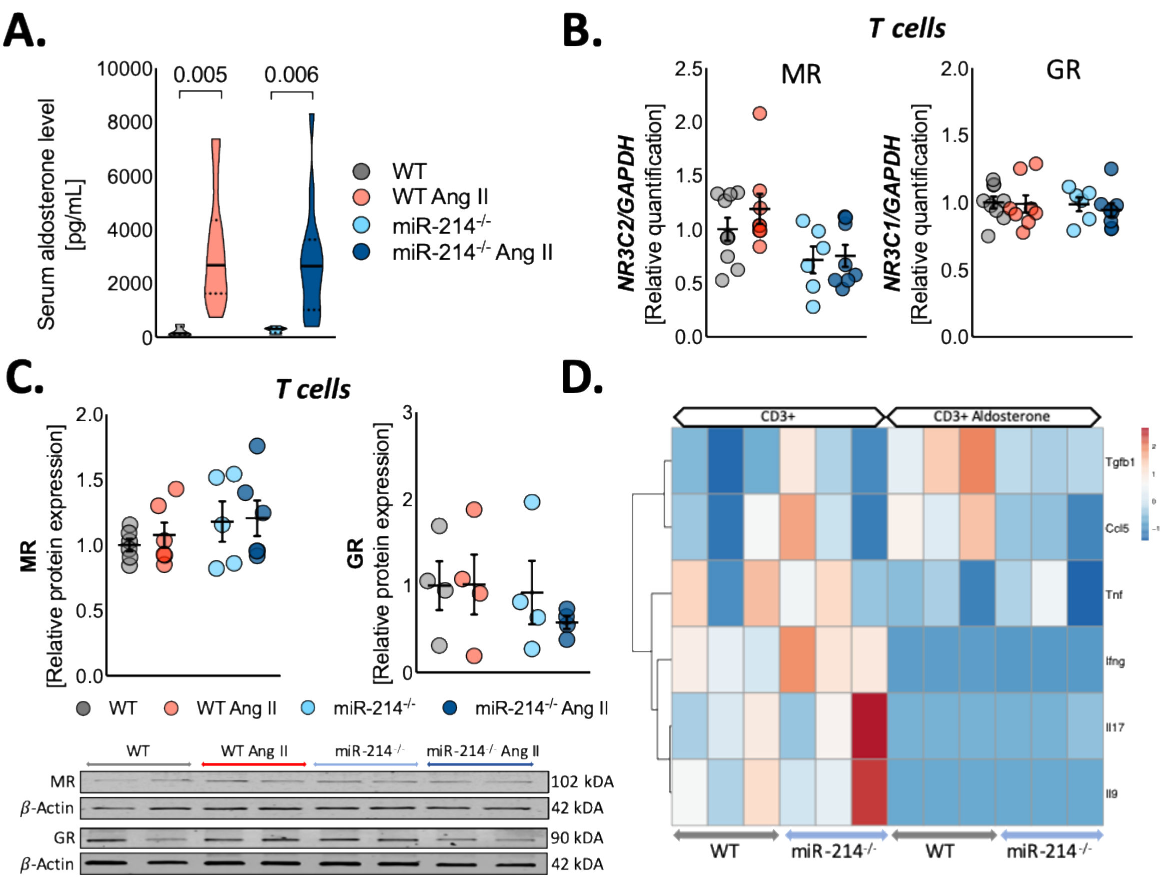

Online Figure V. **Role of miR-214 in the regulation of mineralocorticoid biology A**. Aldosterone levels in urine of WT and miR-214$_{-/-}$ mice infused with sham buffer or Ang II (n=7-13). **B.** mRNA expression levels of mineralocorticoid receptor (MR) (*nr3c2*) and glucocorticoid receptor (GR) (*nr3c1*) in splenic T cells (n=8-9). **C**. Protein level of MR and GR receptors in sorted T cells studied by western blotting (n=6 and n=4, respectively). Data presented as mean±SEM. Data analysed using two-way AOVA with Tukey's test for multiple comparisons (p-values adjusted for n=6). Adjusted p-values indicated **D.** Heat map representing mRNA expression levels (fold change) of key cytokines in WT and miR-214-/- T cells *in vitro* stimulated with anti CD3 in the presence or absence of aldosterone for 24h (n=3). Data analysed using two-way ANOVA with FDR test for multiple comparisons (p-values adjusted for 36 comparisons). Overall p values for two-way ANOVA; Panel **A** (p_{AngII}=2.2e-05, $p_{Genotype}$=0.7, $p_{AngII\ x\ Genotype}$=0.6), Panel **B** for MR (p_{AngII}=0.35, $p_{Genotype}$=5.8e-03, $p_{AngII\ x\ Genotype}$=0.53), for GR MR (p_{AngII}=0.62, $p_{Genotype}$=0.59, $p_{AngII\ x\ Genotype}$=0.78); Panel **C** for MR (p_{AngII}=0.65, $p_{Genotype}$=0.18, $p_{AngII\ x\ Genotype}$=0.82), for GR (p_{AngII}=0.58, $p_{Genotype}$=0.39, $p_{AngII\ x\ Genotype}$=0.55).

Online Figure VI

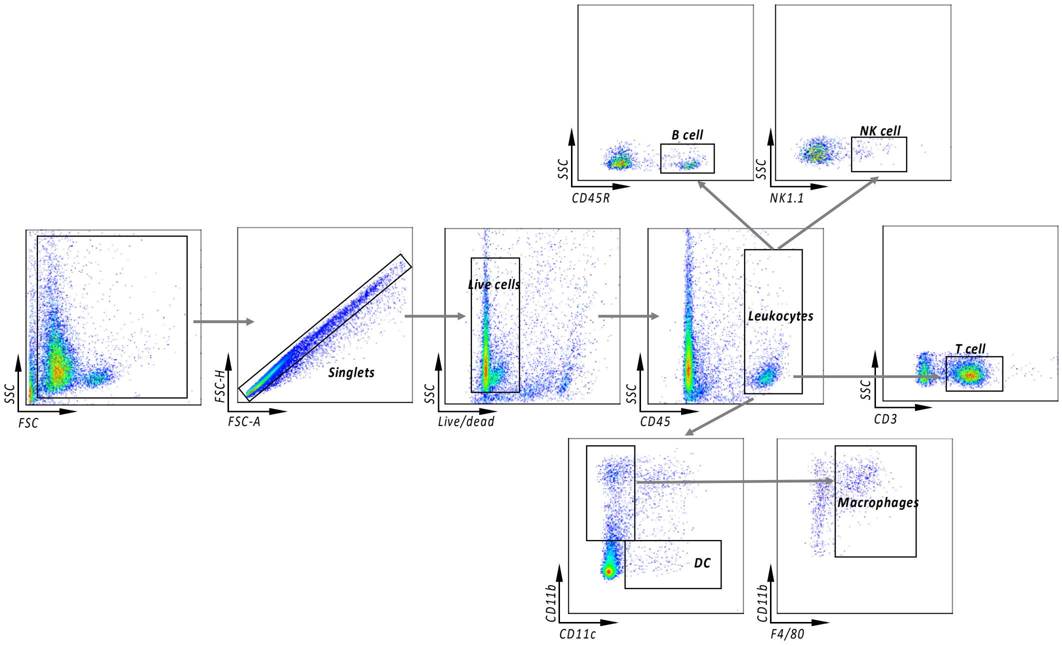

Online Figure VI**. Leukocyte gating strategy for Flow cytometry**.
Cell were first gated by a forward scatter (FSC) and side scatter (SSC) to remove a debris. Next cells were gated for singlets (FSC-H vs. FSC-A) and live cells (SSC vs. Live/Dead Aqua stain) to exclude a dead cell form the analysis. The total leukocytes were gated according CD45 surface expression (SSC vs. CD45). T cells (CD3), B cells (CD45R) and NK cells (NK.1.1) where gated directly form leukocyte gated population (CD45). Dendritic cells were identified by the positive expression of CD11c and lack markers for CD11b within CD45 gate. Macrophages were gated form CD45 and CD11b positive cells according their F4/80 surface marker. Flow cytometry plots for the various markers are shown in bi-exponential format.
