# Supplementary material for: T-Cell–Derived miRNA-214 Mediates Perivascular Fibrosis in Hypertension
Source: Circ Res. 2020 Feb 17;126(8):988–1003. doi: 10.1161/CIRCRESAHA.119.315428 (PMC7147427; doi:10.1161/CIRCRESAHA.119.315428)

Full unedited gel
Figure 3 F.

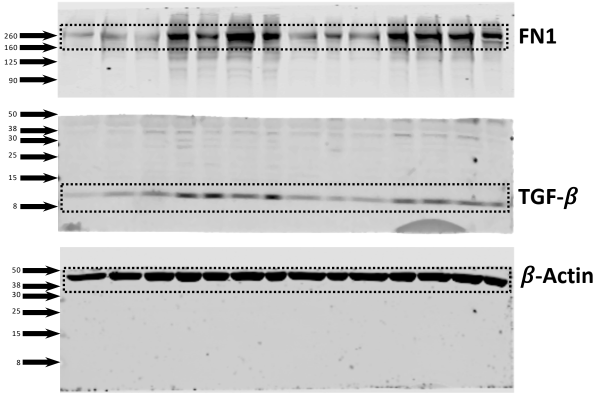

Full unedited gel
Figure 4 F.

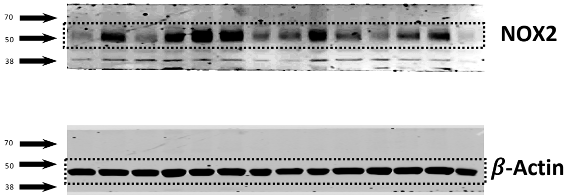

Full unedited gel
Figure 4 C.

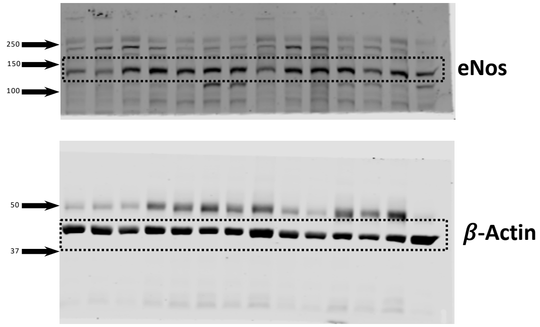

Full unedited gel
Figure 4 F.

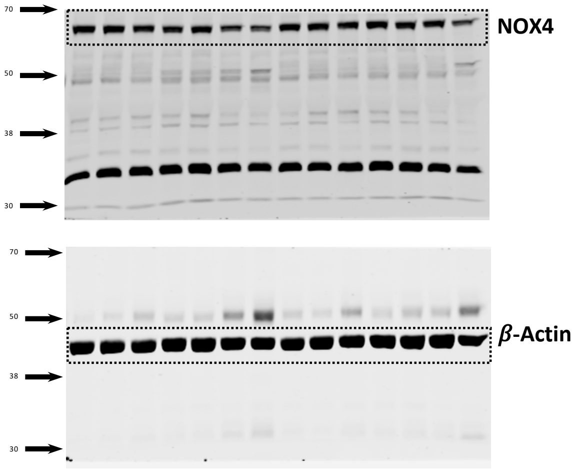

Full unedited gel ***Online Figure V C.***

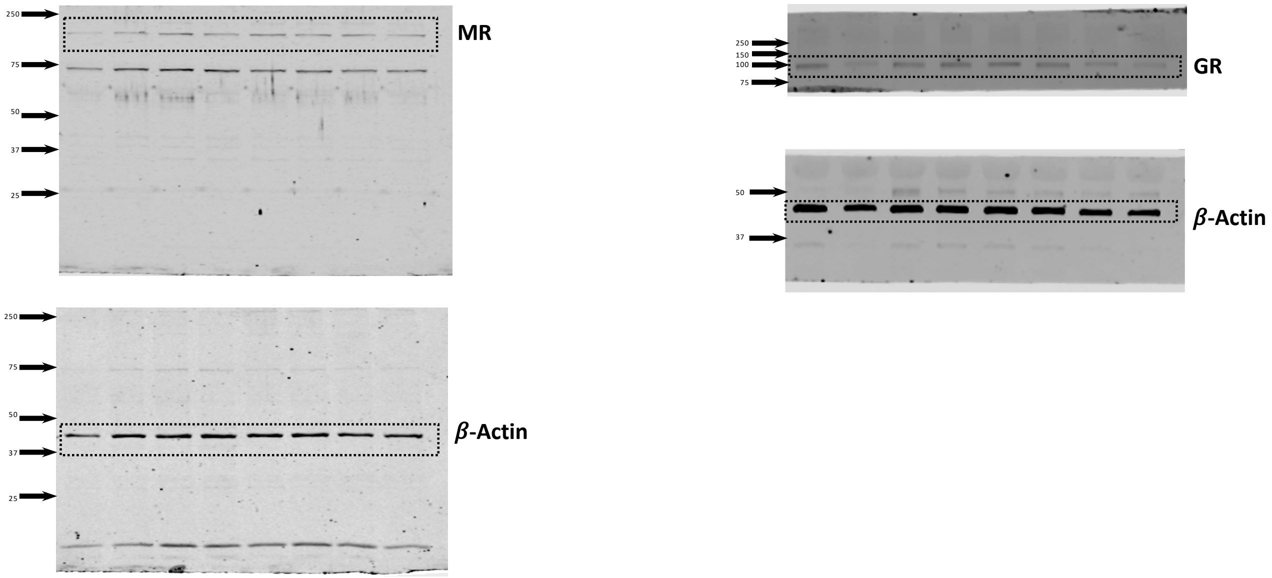

Supplement: Supplementary file 2 [file res-126-988-s002.pdf]
