# Supplementary figures and images for: T-Cell–Derived miRNA-214 Mediates Perivascular Fibrosis in Hypertension
Source: Circ Res. 2020 Feb 17;126(8):988–1003. doi: 10.1161/CIRCRESAHA.119.315428 (PMC7147427; doi:10.1161/CIRCRESAHA.119.315428)

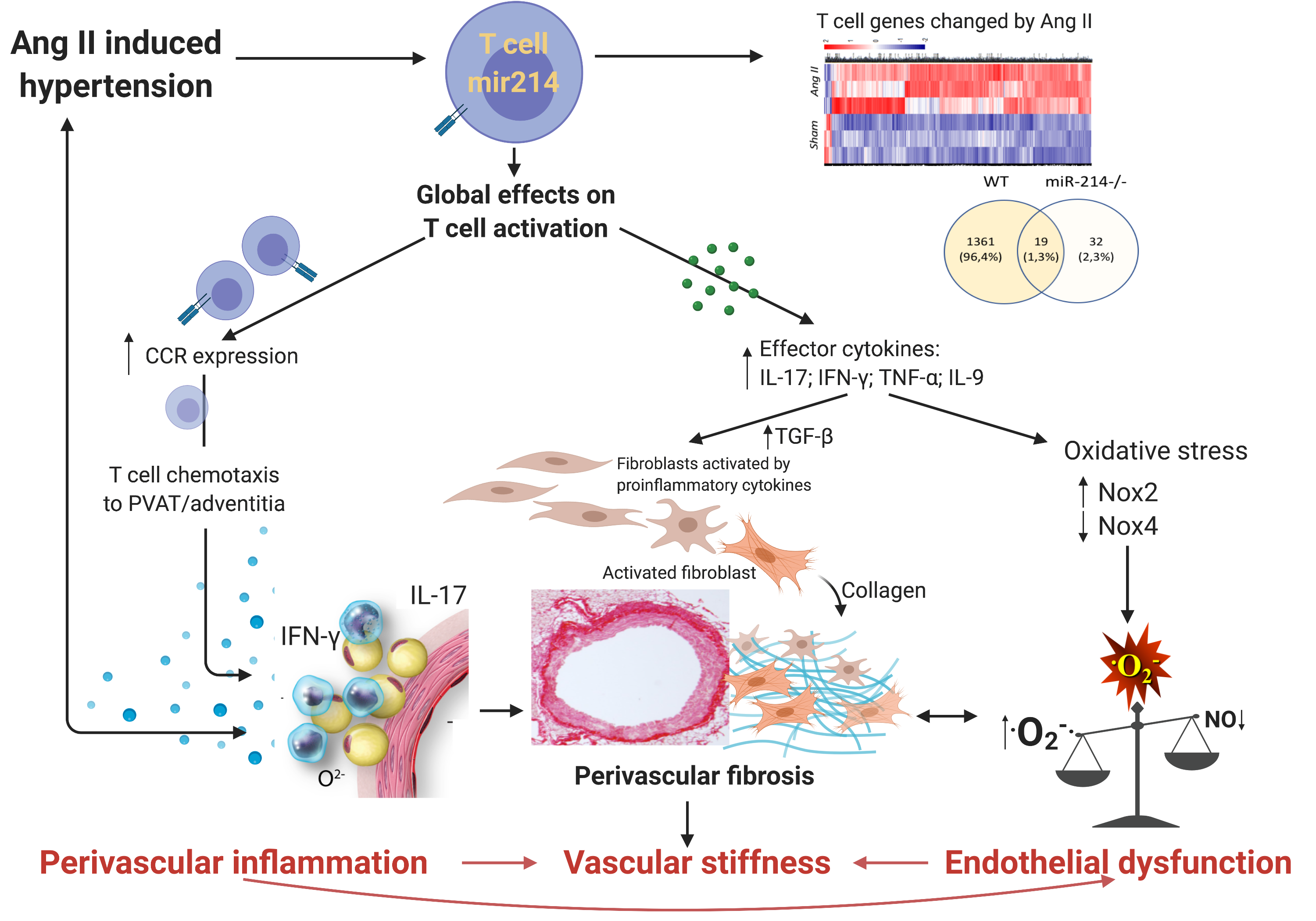

Supplement: Supplementary file 3 [file res-126-988-s003.png]
