# Supplementary material for: T-Cell–Derived miRNA-214 Mediates Perivascular Fibrosis in Hypertension
Source: Circ Res. 2020 Feb 17;126(8):988–1003. doi: 10.1161/CIRCRESAHA.119.315428 (PMC7147427; doi:10.1161/CIRCRESAHA.119.315428)
Supplement: Supplementary file 5 [file res-126-988-s005.pdf]

Major Resources Table

In order to allow validation and replication of experiments, all essential research materials listed in the Methods should be included in the Major Resources Table below. Authors are encouraged to use public repositories for protocols, data, code, and other materials and provide persistent identifiers and/or links to repositories when available. Authors may add or delete rows as needed.

Animals (in vivo studies)

Species	Vendor or Source	Background Strain	Sex	Persistent ID / URL
mouse	Jackson Laboratory	C57BL6	male	https://www.jax.org/strain/000664
mouse	mice were kindly gifted by Dr Eric Olson	miR-214-/- (KO) S129/C57	male	NA
mouse	mice were kindly gifted by Dr Eric Olson	miR-214+/+ (WT) S129/C57	male	NA
mouse	Jackson Laboratory	B6.129S7-*Rag1*^{tm1Mom}/J	male	https://www.jax.org/strain/002216

Genetically Modified Animals

	Species	Vendor or Source	Background Strain	Other Information	Persistent ID / URL
Parent - Male	mouse miR-214-/+	mice were kindly gifted by Dr Eric Olson	S129/C57	NA	NA
Parent - Female	mouse miR-214-/+	mice were kindly gifted by Dr Eric Olson	S129/C57	NA	NA
Rag1$^{-/-}$	mouse	Jackson Laboratory	B6.129S7-*Rag1*^{tm1Mom}/J	male	https://www.jax.org/strain/002216

Antibodies

Target antigen	Vendor or Source	Catalog #	Working concentration	Persistent ID / URL
CD45 (30-F11)	BioLegend	#103108	1:200	https://www.biolegend.com/en-us/products/fitc-anti-mouse-cd45-antibody-99
CD3 (145-2C11)	BioLegend	#100320	1:100	https://www.biolegend.com/en-us/products/pe-cy7-anti-mouse-cd3epsilon-antibody-1899
CD11b (M1/70)	BioLegend	#101208	1:80	https://www.biolegend.com/en-us/products/pe-anti-mouse-human-cd11b-antibody-349
F4/80 (BM8)	BioLegend	#123130	1:50	https://www.biolegend.com/en-us/products/alexa-fluor-700-anti-mouse-f4-80-antibody-6556
CD11c (N418)	BioLegend	#117316	1:80	https://www.biolegend.com/en-us/products/pe-cy5-anti-mouse-cd11c-antibody-3085
B220 (RA3-6B2)	BioLegend	#103212	1:80	https://www.biolegend.com/en-us/products/apc-anti-mouse-human-cd45r-b220-antibody-442
NK1.1 (PK136)	BioLegend	#108726	1:20	https://www.biolegend.com/en-us/products/percp-anti-mouse-nk-1-1-antibody-4288
CD3 (145-2C11)	BioLegend	#100312	1:100	https://www.biolegend.com/en-us/products/apc-anti-mouse-cd3epsilon-antibody-21
CCR2 (SA203G11)	BioLegend	#150608	1:50	https://www.biolegend.com/en-us/products/fitc-anti-mouse-cd192-ccr2-antibody-13354
CCR5 (HM-CCR5)	eBioscience	#12-1951-83	1:50	https://www.thermofisher.com/antibody/product/CD195-CCR5-Antibody-clone-HM-CCR5-7A4-Monoclonal/12-1951-82
CCR1 (S15040E)		#152503	1:50	https://www.biolegend.com/en-us/products/apc-anti-mouse-cd191-ccr1-antibody-13643
CXCR3 (CXCR3-173)	BioLegend	#126542	1:100	https://www.biolegend.com/en-us/products/alexa-fluor-488-anti-mouse-cd183-cxcr3-antibody-14075
CCR6 (29-2L17)	BioLegend	#129815	1:50	https://www.biolegend.com/en-us/products/pe-cy7-anti-mouse-cd196-ccr6-antibody-6152
CCR4 (2G12)	BioLegend	#131204	1:50	https://www.biolegend.com/en-us/products/pe-anti-mouse-cd194-ccr4-antibody-5379
IFN (XMG1.2)	BioLegend	#505813	1:100	https://www.biolegend.com/en-us/products/alexa-fluor-488-anti-mouse-ifn-gamma-antibody-2721

DOI [to be added]

TNF-α (MP6 XT22)	BD Bioscience	#554419	1:100	https://www.bdbiosciences.com/us/applications/research/t-cell-immunology/th-1-cells/intracellular-markers/cytokines-and-chemokines/mouse/pe-rat-anti-mouse-tnf-mp6-xt22/p/554419
IL-17a (TC11-18H10.1)	BioLegend	#506904	1:100	https://www.biolegend.com/en-us/products/pe-anti-mouse-il-17a-antibody-1633
IL-9 (RM9A4,)	BioLegend	#514111	1:300	https://www.biolegend.com/en-us/products/percp-cy5-5-anti-mouse-il-9-antibody-9037
CD45	BioLegend	#103132	1:200	https://www.biolegend.com/en-us/products/percpcyanine55-anti-mouse-cd45-antibody-4264
anti-DIG antibody	Roche	#11093274910	1:500	https://www.sigmaaldrich.com/catalog/product/roche/11093274910?lang=en&region=GB
Nox2	BD	#611414	1:2000	https://www.bdbiosciences.com/us/reagents/research/antibodies-buffers/cell-biology-reagents/cell-biology-antibodies/purified-mouse-anti-gp91phox-53gp91phox/p/611414
Nox4	Novus	#NB110-58849	1:1000	https://www.novusbio.com/products/nox4-antibody_nb110-58849
eNOS	BD	#610297	1:2000	https://www.bdbiosciences.com/eu/reagents/research/antibodies-buffers/cell-biology-reagents/cell-biology-antibodies/purified-mouse-anti-enosnos-type-iii-3enosnos-type-iii/p/610297
TGF-β	Cell Signaling	#3711S	1:1000	https://www.cellsignal.co.uk/products/primary-antibodies/tgf-b-antibody/3711?site-search-type=Products&N=4294956287&Ntt=3711s&fromPage=plp&_requestid=2123004
FN1	Sigma-Aldrich	#F3648	1:1000	https://www.sigmaaldrich.com/catalog/product/sigma/f3648?lang=en&region=GB
MRC	Santa Cruz Biotechnology	#sc-71554	1:500	https://www.scbt.com/p/mcr-antibody-4i342
GR	Santa Cruz Biotechnology	#sc-1004	1:500	https://www.scbt.com/p/gr-antibody-m-20
beta-actin	Abcam	#ab8227	1:1000	https://www.abcam.com/beta-actin-antibody-ab8227.html
IRDye®800 CW (anti-mouse or anti-rabbit)	LI-COR	#925-32210 Or #925-32211	1:15000	https://www.licor.com/bio/reagents/irdye-800cw-secondary-antibodies
IRDye®680 LT (anti-mouse or anti-rabbit)	LI-COR	#925-68070 Or #925-68071	1:15000	https://www.licor.com/bio/reagents/irdye-680lt-secondary-antibodies

DNA/cDNA Clones

Clone Name	Sequence	Source / Repository	Persistent ID / URL
SNORNA202	TaqMan™	Thermo-Fisher	001232
U6	TaqMan™	Thermo-Fisher	001973
miR-214-3p	TaqMan™	Thermo-Fisher	002306
miR-214-5p	TaqMan™	Thermo-Fisher	002293
miR-39 *C. elegans*	TaqMan™	Thermo-Fisher	000200
miR-199a-5p	TaqMan™	Thermo-Fisher	000498
mir-199a-3p	TaqMan™	Thermo-Fisher	002304
Pri-miR-214	TaqMan™	Thermo-Fisher	Mm03307122_pri
Col1a1	TaqMan™	Thermo-Fisher	Mm00801666_g1
Col3a1	TaqMan™	Thermo-Fisher	Mm00802300_m1
Col5a1	TaqMan™	Thermo-Fisher	Mm00489299_m1
Cd3e	TaqMan™	Thermo-Fisher	Mm01179194_m1
Tgfb1	TaqMan™	Thermo-Fisher	Mm01178820_m1
tnf	TaqMan™	Thermo-Fisher	Mm00443258_m1

DOI [to be added]

Il17	TaqMan™	Thermo-Fisher	Mm00439619_m1
Il9	TaqMan™	Thermo-Fisher	Mm00434305_m1
Ccl5	TaqMan™	Thermo-Fisher	Mm01302428_m1
ifng	TaqMan™	Thermo-Fisher	Mm00801778_m1
Nox2	TaqMan™	Thermo-Fisher	Mm01287743_m1
Nox4	TaqMan™	Thermo-Fisher	Mm00479246_m1
Fn1	TaqMan™	Thermo-Fisher	Mm01256744_m1
Nr3c1	TaqMan™	Thermo-Fisher	Mm00433832_m1
Nr3c2	TaqMan™	Thermo-Fisher	Mm01241596_m1

Cultured Cells

Name	Vendor or Source	Sex (F, M, or unknown)	Persistent ID / URL
Primary VSMCs	C56BL6 mouse	Male	NA
Primary fibroblasts	C56BL6 mouse	Male	NA
THP-1	Sigma-Aldrich	unknown	https://www.sigmaaldrich.com/catalog/product/sigma/cb_88081201?lang=en&region=GB
Endothelial cells	Thermo-Fisher	unknown	https://www.thermofisher.com/order/catalog/product/C0105C?SID=srch-hj-C0105C#/C0105C?SID=srch-hj-C0105C

Data & Code Availability

Description	Source / Repository	Persistent ID / URL
RNA-seq datasets	GSE143809	https://www.ncbi.nlm.nih.gov/geo/query/acc.cgi?acc=GSE143809

Other

Description	Source / Repository	Persistent ID / URL
EasySepTM Mouse T cell Isolation Kit	STEMCELL Technologies	19851
Angiotensin II	Sigma-Aldrich	A2900
hydrochlorothiazide	Sigma-Aldrich	H4759
hydralazine	Sigma-Aldrich	H1753
Weigert's iron hematoxylin solution	Sigma-Aldrich	HT1079-1SET
Trichrome (Masson) stain Kit	Sigma-Aldrich	HT15
phorbol 12-myristate13-acetate (PMA) with ionomycin	eBioscience	00-4970-93
mirVana kit	Ambion	AM1560
Megaplex primmer pools and profiled with TaqMan Rodent MicroRNA A Array v.2.0	Applied Biosystems)	4398967
High Capacity cDNA reverse kit	Applied Biosystems	4374967
miRNeasy mini kit	Qiagen	217004
MiRNeasy serum/plasma kit	Qiagen	217184
miRNeasy/Serum/Plasma Spike-In Control	Qiagen	219610
TaqMan miRNA Reverse transcription kit	ThermoFisher	4366597
proteinase K	Life Technologies	25530049
miR-214-3p or scramble probe labeled with 3′ and 5′ digoxigenin	DIG, Exiqon	YD00611471 and YD00699004
BM purple solution	Roche	11442074001
Collagenase II	Sigma-Aldrich	C6885
Elastase	Sigma-Aldrich	E7885
Soybean Trypsin Inhibitor	Sigma-Aldrich	T9003
IFN-γ	PeproTech	300-02
LPS	Sigma-Aldrich	L2887
IL-4	PeproTech	200-04

DOI [to be added]

IL-13	PeproTech	200-13
CXCL10	PeproTech	250-16
CCL5	PeproTech	250-07
IL-17	R&D Systems	421-ML
TNFα	R&D Systems	410-MT
TGFβ	R&D Systems	7666-MB
ET-1	R&D Systems	1160
aldosterone	Sigma-Aldrich	A9477
RIPA lysis and extraction buffer	Thermo Scientific	89900
HaltTM Protease and Phosphatase Inhibitors	Thermo Scientific	78440
Aldosterone Assay	R&D	KGE016
Liberase	Roche	5401020001
eFluorTM 506	eBioscience	65-0866-14
Zombie Aqua™ Fixable Viability Kit	BioLegend	423101
RPMI 1640	Gibco	61870044
OCT	Tissue-Tek®	4583

DOI [to be added]
