# Supplementary material for: T-Cell–Derived miRNA-214 Mediates Perivascular Fibrosis in Hypertension
Source: Circ Res. 2020 Feb 17;126(8):988–1003. doi: 10.1161/CIRCRESAHA.119.315428 (PMC7147427; doi:10.1161/CIRCRESAHA.119.315428)
Supplement: Supplementary file 6 [file res-126-988-s006.pdf]

Circulation Research
T cell-derived miRNA-214 mediates perivascular fibrosis in hypertension.
Corresponding Author: Prof. Tomasz J. Guzik

*** Long In Vivo Checklist**
Circulation Research - Preclinical Animal Testing: A detailed checklist has been developed as a prerequisite for every publication involving preclinical studies in animal models. ***Checklist items must be clearly described in the manuscript; if the answer to a question is "No", an explanation should be provided both within the manuscript text and on the following screen.*** *If this information (checklist items and/or explanations) cannot be included in the main manuscript because of space limitations, please include it in an online supplement. If the manuscript is accepted, this checklist will be published as an online supplement. See the explanatory editorial for further information.*

This study involves use of animal models:
Yes

Study Design

The experimental group(s) have been clearly defined in the article, including number of animals in each experimental arm of the study.	Yes
An overall study timeline is provided.	Yes
The protocol was prospectively written	Yes
The primary and secondary endpoints are specified	Yes
For primary endpoints, a description is provided as to how the type I error multiplicity issue was addressed (e.g., correction for multiple comparisons was or was not used and why). (Note: correction for multiple comparisons is not necessary if the study was exploratory or hypothesis-generating in nature).	Yes
A description of the control group is provided including whether it matched the treated groups.	Yes

Inclusion and Exclusion criteria

Inclusion and exclusion criteria for enrollment into the study were defined and are reported in the manuscript.	No
These criteria were set *a priori* (before commencing the study).	No

Randomization

Animals were randomly assigned to the experimental groups.	Yes
If random assignment was not used, adequate explanation has been provided.	N/A
Type and methods of randomization have been described.	Yes
Allocation concealment was used.	Yes
Methods used for allocation concealment have been reported.	Yes

Blinding

Blinding procedures with regard to masking of group/treatment assignment from the experimenter were used and are described. The rationale for nonblinding of the experimenter has been provided, if such was not performed.	Yes
Blinding procedures with regard to masking of group assignment during outcome assessment were used and are described.	Yes
If blinding was not performed, the rationale for nonblinding of the person(s) analyzing outcome has been provided.	No

Sample size and power calculations

Formal sample size and power calculations were conducted before commencing the study based on *a priori* determined outcome(s) and treatment effect(s), and the data are reported.	Yes
If formal sample size and power calculation was not conducted, a rationale has been provided.	N/A

Data Reporting

Baseline characteristics (species, sex, age, strain, chow, bedding, and source) of animals are reported.	Yes
The number of animals in each group that were randomized, tested, and excluded and that died is reported. If the experimentation involves repeated measurements, the number of animals assessed at each time point is provided is provided for all experimental groups.	N/A
Baseline data on assessed outcome(s) for all experimental groups are reported.	N/A
Details on important adverse events and death of animals during the course of the experiment are reported for all experimental groups.	N/A
Numeric data on outcomes are provided in the text or in a tabular format in the main article or as supplementary tables, in addition to the figures.	N/A
To the extent possible, data are reported as dot plots as opposed to bar graphs, especially for small sample size groups.	Yes
In the online Supplemental Material, methods are described in sufficient detail to enable full replication of the study.	Yes

Statistical methods

The statistical methods used for each data set are described.	Yes
For each statistical test, the effect size with its standard error and *P* value is presented. Authors are encouraged to provide 95% confidence intervals for important comparisons.	No
Central tendency and dispersion of the data are examined, particularly for small data sets.	Yes
Nonparametric tests are used for data that are not normally distributed.	Yes
Two-sided *P* values are used.	Yes
In studies that are not exploratory or hypothesis-generating in nature, corrections for multiple hypotheses testing and multiple comparisons are performed.	Yes
In "negative" studies or null findings, the probability of a type II error is reported.	No

Experimental details, ethics, and funding statements

Details on experimentation including formulation and dosage of therapeutic agent, site and route of administration, use of anesthesia and analgesia, temperature control during experimentation, and postprocedural monitoring are described.	Yes
Both male and female animals have been used. If not, the reason/justification is provided.	No
Statements on approval by ethics boards and ethical conduct of studies are provided.	Yes
Statements on funding and conflicts of interests are provided.	Yes

---

Date completed: 02/08/2020 13:50:48
User pid: 23565
